# Targeting mTOR to overcome resistance to hormone and CDK4/6 inhibitors in ER-positive breast cancer models

**DOI:** 10.1038/s41598-023-29425-y

**Published:** 2023-02-15

**Authors:** María Jimena Rodriguez, María Cecilia Perrone, Marina Riggio, Marta Palafox, Valeria Salinas, Andrés Elia, Natali Daiana Salgueiro, Andrea Eugenia Werbach, María Paula Marks, Marcelo A. Kauffman, Luciano Vellón, Violeta Serra, Virginia Novaro

**Affiliations:** 1 Instituto de Biología y Medicina Experimental (IBYME-CONICET), Protein Kinases and Cancer Laboratory, ADN1428 CABA, Buenos Aires, Argentina; 2 Vall D’Hebron Institute of Oncology (VHIO), Experimental Therapeutics Group, 08035 Barcelona, Spain; 3 Hospital JM Ramos Mejía, Neurogenetics Unit, C1221ADC CABA, Buenos Aires, Argentina; 4 Universidad Austral, Translational Medicine Research Institute IIMT-CONICET, B1630FHB Pilar, Buenos Aires, Provincia de Buenos Aires Argentina; 5 Instituto de Biología y Medicina Experimental (IBYME-CONICET), Stem Cells Laboratory, ADN1428 CABA, Buenos Aires, Argentina

**Keywords:** Cancer, Cell biology

## Abstract

Resistance to therapy remains a major obstacle in cancer management. Although treatment with hormone and CDK4/6 inhibitors is successful in luminal breast cancer, resistance to these treatments is frequent, highlighting the need for novel therapeutic strategies to delay disease progression and improve patient survival. Here, we assessed the mechanisms of acquired resistance using T47D and MCF-7 tamoxifen- and palbociclib-resistant cell-line variants in culture and as xenografts, and patient-derived cells (PDCs) obtained from sensitive or resistant patient-derived xenografts (PDXs). In these models, we analyzed the effect of specific kinase inhibitors on survival, signaling and cellular aggressiveness. Our results revealed that mTOR inhibition is more effective than PI3K inhibition in overcoming resistance, irrespective of *PIK3CA* mutation status, by decreasing cell proliferation and tumor growth, as well as reducing cell migration and stemness. Moreover, a combination of mTOR and CDK4/6 inhibitors may prevent pathway reactivation downstream of PI3K, interfering with the survival of resistant cells and consequent tumor escape. In conclusion, we highlight the benefits of incorporating mTOR inhibitors into the current therapy in ER + breast cancer. This alternative therapeutic strategy not only enhances the antitumor response but may also delay the emergence of resistance and tumor recurrence.

## Introduction

Estrogen receptor (ER)-positive breast cancers account for approximately 70% of all breast cancer cases. Although the use of hormone inhibitors, such as tamoxifen, fulvestrant, and aromatase inhibitors, has remained the mainstay of ER + breast cancer treatment, the emergence of resistance has led to the addition of CDK4/6 inhibitors to this therapy. Three CDK4/6 inhibitors, palbociclib, ribociclib, and abemaciclib, have shown improved clinical outcomes in patients with advanced or metastatic ER + breast cancer and were therefore approved for use in combination with endocrine therapy in first- or second-line settings^1^. Despite the benefits of this approach, patients can ultimately undergo disease progression^2^, emphasizing the importance of unraveling the mechanisms involved in resistance to these inhibitors to find alternative targets.

Numerous mechanisms are involved in the development of endocrine and CDK4/6 inhibitor resistance in breast and other types of cancer^3–6^**,** including downregulation of nuclear ER, mutation of ESR1 gene, dysregulation of the cyclin D1/CDK4/6/Rb axis and PKCα, as well as genetic and epigenetic alterations in survival pathways, such as RAS/MAPK/ERK and PI3K/AKT/mTOR. Notably, most breast tumors present alterations in different components of the PI3K pathway, with the most frequent being PTEN loss and activating mutations in *PIK3CA*, which encodes the catalytic subunit alpha of PI3K^7,8^.

Hyperactivation of the PI3K/AKT/mTOR pathway influences tumorigenesis, aggressiveness, the immune microenvironment, and drug response^9–11^. PI3K activation leads to the regulation of several downstream molecules, including mTOR, which, in turn, promotes the phosphorylation and activation of ribosomal protein S6. Phosphorylated S6 (pS6) has been associated with neoadjuvant therapeutic response in breast cancer models^12^, endocrine therapy resistance in patients with ER + breast tumors^13^ and earlier recurrence^14^.

Several PI3K/AKT/mTOR inhibitors have been evaluated for the treatment of refractory ER + breast cancer. Everolimus was the first mTOR inhibitor approved for ER + HER2- patients with advanced breast cancer who relapsed to hormone therapy^15^. More recently, the PI3Kα inhibitor alpelisib has been approved for patients with ER + HER2-*PIK3CA*-mutated advanced or metastatic breast cancer^16,17^. Preclinical studies have demonstrated the effectiveness of PI3K/AKT/mTOR inhibitors in overcoming or delaying tumor resistance to CDK4/6 inhibitors^18–20^. Moreover, the combined inhibition of PI3K/AKT/mTOR and CDK4/6 is currently being evaluated in clinical trials for advanced or metastatic breast cancer, as well as the use of PI3K/AKT/mTOR and hormone inhibitors in patients who relapse to CDK4/6 inhibitors (NCT03056755).

The crosstalk between the PI3K/AKT/mTOR pathway and cyclin D1/CDK4/6/Rb axis occurs at several nodes. For instance, the cyclin D1/CDK4/6 complex controls cell cycle progression not only directly through Rb phosphorylation but also by promoting mTORC1 activation and S6 phosphorylation, further stimulating protein synthesis and cell growth^21–23^. On the other hand, some studies have shown that hyperphosphorylated Rb binds to Sin1, inhibiting mTORC2-mediated activation of AKT^24^. Overall, evidence suggests that cross-regulation between these two pathways could be cell-context-dependent and may influence the efficiency of selective inhibitors targeting different levels.

In this study, we developed tamoxifen (TR), palbociclib (PR), and tamoxifen + palbociclib (TPR) resistant variants from T47D and MCF-7 ER + breast cancer cell lines to elucidate the mechanisms involved in tumor resistance and explore novel potential targets. Since CDK4/6 inhibitors are typically indicated for patients who relapse after hormonal therapy, we developed a dual-resistance model (TPR) that better represents this treatment sequence. To complement the studies in cell lines, we analyzed patient-derived tumor cells (PDCs) obtained from sensitive or resistant ER + patient-derived xenografts (PDXs).

Collectively, our findings indicate that targeting mTOR rather than PI3K is more effective in reducing cell proliferation, tumor growth, cell migration, and stemness, independent of *PIK3CA* mutation status. Furthermore, the incorporation of mTOR inhibitors into the therapy with CDK4/6 inhibitors may abrogate or delay the onset of drug resistance, providing a higher therapeutic effect in both tamoxifen- and palbociclib-resistant settings.

## Results

### Long-term treatment with tamoxifen and palbociclib leads to the downregulation of hormone receptors and alterations in the cell cycle

**Figure 1:**
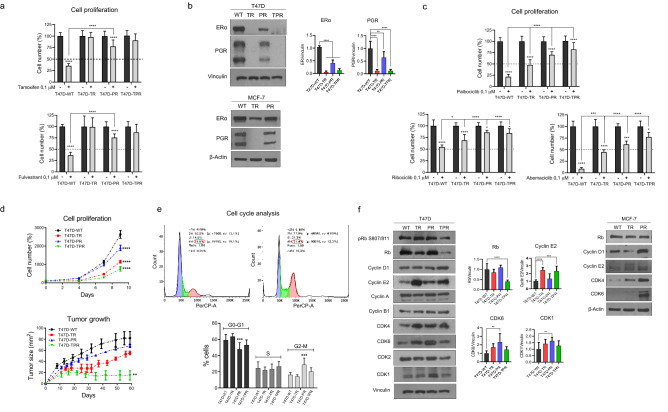
The resistant cells display alterations in proliferation and cell cycle. (**a**) Effect of ER inhibitors on cell proliferation. T47D cell variants were treated with 4-hydroxytamoxifen (0.1 µM), fulvestrant (0.1 µM) or vehicle for 7 days and counted at the end of the experiment. ****p < 0.0001. Data represent mean ± SD, two-way ANOVA followed by Tukey's test (independent replicates n = 3, with 4 experimental replicates in each group). (**b**) Expression of hormone receptors. Protein lysates were obtained under basal conditions and analyzed by immunoblot with the indicated antibodies. For T47D variants, bands were quantified by densitometry and relativized to their loading control. **p < 0.01, ****p < 0.0001. Data represent mean ± SD, one-way ANOVA followed by Dunnett’s test (independent replicates n = 3, with 3 experimental replicates in each group). (**c**) Effect of CDK4/6 inhibitors on cell proliferation. T47D cells were treated with palbociclib (0.1 µM), ribociclib (0.1 µM), abemaciclib (0.1 µM) or vehicle for 7 days and counted at the end of the treatment. *p < 0.05, ***p < 0.001, ****p < 0.0001. Data represent mean ± SD, two-way ANOVA followed by Tukey's test (independent replicates n = 3, with 4 experimental replicates in each group). (**d**) Cell proliferation and tumor growth. Top: Cells were cultured in the absence of drugs and quantified every 2 days. ****p < 0.0001. Data represent mean ± SD, two-way ANOVA followed by Dunnett's test (independent replicates n = 3, with 4 experimental replicates in each group). Bottom: Tumor growth curves. 8 × 10^6^ cells of each variant were injected subcutaneously in the lateral flank of NSG mice, previously implanted with silastic pellets containing 17β-estradiol (0.25 mg). **p < 0.01. Data represent mean ± SD, one-way ANOVA followed by Dunnett's test at the end of the experiment (independent replicates n = 2, with 3 experimental replicates in each arm of treatment). Representative curve of two. (**e**) Cell cycle analyses. T47D cells were stained with propidium iodide and analyzed by flow cytometry. ***p < 0.001. Data represent mean ± SD, one-way ANOVA followed by Dunnett's test (independent replicates n = 4, with 4 experimental replicates in each group). (**f**) Expression of cell cycle proteins. Protein lysates were obtained under basal conditions and analyzed by immunoblot with the indicated antibodies. For T47D variants, bands were quantified by densitometry and relativized to their loading control. **p < 0.01, ***p < 0.001, ****p < 0.0001. Data represent mean ± SD, one-way ANOVA followed by Dunnett's test (independent replicates n = 2, with 3 experimental replicates in each group). Original blots/gels are presented in Supplementary Material, unprocessed western blots section.

Tamoxifen (TR), palbociclib (PR), and double-resistant (TPR) cell variants were generated from T47D and MCF-7 wild-type (WT) cell lines, as described in Materials and Methods. Resistance to these agents was confirmed by proliferation assays. Treatment with the hormone inhibitors tamoxifen and fulvestrant had no significant impact on the proliferation of tamoxifen- and double-resistant cells, whereas palbociclib-resistant cells showed reduced sensitivity to these agents compared to parental T47D-WT cells (Fig. 1a). It is worthwhile mentioning that MCF-7-TPR cells still respond to palbociclib despite 12 months of treatment (Supplementary Fig. S1a). These results are consistent with a substantial decrease in ER and progesterone receptor (PGR) expression in T47D and MCF-7 resistant models (Fig. 1b). Additionally, all resistant variants presented decreased sensitivity to palbociclib compared to T47D-WT, including the tamoxifen-resistant cells (Fig. 1c). Furthermore, the resistant variants also showed cross-resistance to other CDK4/6 inhibitors such as ribociclib and abemaciclib. Remarkably, T47D-TR and T47D-PR xenografts maintained their resistance to tamoxifen and palbociclib, respectively (Supplementary Fig. S1b).

All T47D-resistant cells showed lower proliferation rates in cell culture as well as decreased tumor growth in vivo compared to parental cells (Fig. 1d). In particular, the double-resistant cells formed small tumors that were unable to grow further. Notably, estradiol supplementation was necessary for tumor growth in all cell lines (Supplementary Fig. S1c). The xenograph study was only attempted in T47D variants.

Next, we performed cell-cycle phase analysis to determine whether the cells were arrested in any particular phase. Surprisingly, only the palbociclib-resistant variant presented a higher percentage of cells in the G2/M phase, with a simultaneous decrease in the proportion of cells in G0/G1 (Fig. 1e). Given that palbociclib triggers cell cycle arrest in G0/G1^25^, we speculate that T47D-PR cells were able to bypass this arrest and resume cell cycle progression, accumulating in G2/M. Overall, no significant number of aberrant mitoses was observed in this resistant variant (Supplementary Fig. S1d).

To decipher the mechanisms by which cells acquired resistance, we analyzed the expression of several cell cycle proteins (Fig. 1f). A significant increase in cyclin E2 (T47D-TR and T47D-TPR) as well as in CDK6 and CDK1 levels (T47D-PR) was observed, along with a decrease in Rb (T47D-TPR), compared to the parental cells. When analyzing MCF-7 variants, we found higher levels of cyclin D1, CDK4, and CDK6 in MCF-7-PR cells. This upregulation of cyclins and CDKs may compensate for the inhibitory effect of the drugs on the cell cycle.

### Tamoxifen and palbociclib-resistant cells exhibit an altered phenotype

**Figure 2:**
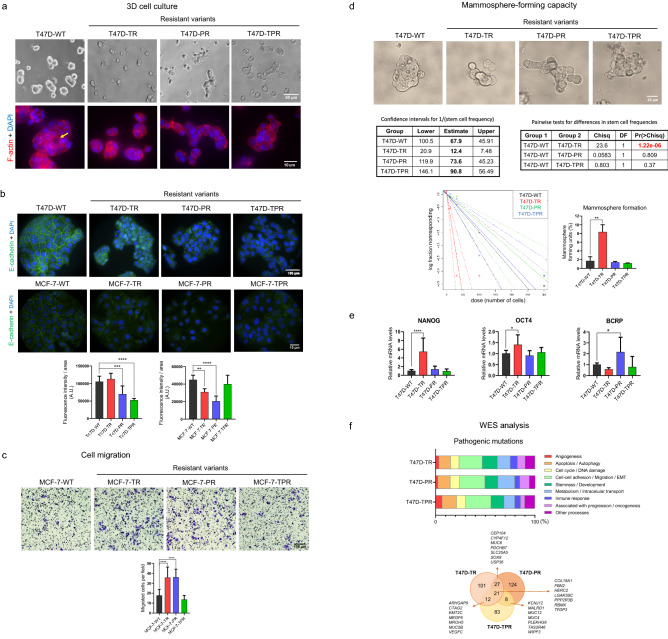
The resistant cells exhibit an altered phenotype. (**a**) 3D cultures. T47D cells were cultured on Geltrex for 48 h. 3D cultures were stained for F-actin with phalloidin (red), and nuclei were counterstained with DAPI (blue). Some of the T47D-WT spheroids exhibited a lumen (yellow arrow). (**b**) Expression and localization of E-cadherin. Confocal microscopy images of immunofluorescence staining for E-cadherin (green). Nuclei were counterstained with DAPI (blue). **p < 0.01, ***p < 0.001. Data represent mean ± SD, one-way ANOVA followed by Dunnett's test (independent replicates n = 2, with 10 analyzed fields in each group). Since resistant cells are bigger than wild type cells, fluorescence intensity was adjusted to cell area. (**c**) Cell migration. Transwell migration assays were performed by seeding MCF-7 cells onto 8 µm-pore inserts in the presence of a serum gradient. After 24 h, cells that passed through the insert and attached to the other side were quantified. ***p < 0.001. Data represent mean ± SD, one-way ANOVA followed by Dunnett's test (independent replicates n = 3, with 4 experimental replicates in each group). (**d**) Mammosphere-forming capacity. Mammosphere formation assays and Extreme limiting dilution assay were performed. Sphere formation frequencies were compared using the ELDA web tool (left). The number of cells seeded per well versus the logarithmic fraction of wells without any detected spheres was plotted (middle). Trend lines represent the estimated active cell frequency, dotted lines show the 95% confidence interval. Percentage of sphere-forming units was plotted according to % = 1/Frequency × 100 (right). **p < 0.01. Data represent mean ± SD, one-way ANOVA followed by Dunnett's test (independent replicates n = 2, with 6 experimental replicates in each group). (**e**) Expression of stemness markers. mRNA levels of NANOG, OCT4 and BCRP were measured by qRT-PCR. Expression levels were normalized to the GAPDH expression for each variant. *p < 0.05, ****p < 0.0001. Data represent mean ± SD, one-way ANOVA followed by Dunnett's test (independent replicates n = 3, with 3 experimental replicates in each group). (**f**) Pathogenic mutations in T47D-resistant cells. Mutations that were not present in the parental cells were filtered by potential pathogenicity using REVEL, SIFT, Polyphen2 and CLNSIG predictors. Top: Mutated genes were grouped according to their function or the cellular processes they are involved in. Bottom: Venn Diagram showing the number of pathogenic mutations in the resistant cells and some relevant mutated genes. Original images are presented in Supplementary Material, unprocessed photomicrographs section.

Some phenotypic changes occurred during the development of resistance, as evidenced by the increased heterogeneity in cell size and morphology compared to the parental cells (Supplementary Fig. S1e). When grown on the basement membrane matrix Geltrex, the parental cells formed large compact spheroids, some of which even displayed a lumen. However, the resistant variants formed smaller (T47D-TR) or disorganized structures with grape-like morphology (T47D-PR and T47D-TPR) (Fig. 2a), which has been associated with decreased cell–cell junctions and a more invasive phenotype^26^. Reduced expression or aberrant (nuclear and cytoplasmic) localization of E-cadherin was observed in both T47D- and MCF-7-resistant cells, except for MCF-7-TPR cells (Fig. 2b). This correlates with the increased migratory capacity of MCF-7-TR and MCF-7-PR variants, as evidenced by transwell assays (Fig. 2c).

Previous studies have suggested that resistance to endocrine and chemotherapy might be associated with enrichment in stem cells, as they exhibit lower sensitivity to treatments^27,28^**.** To assess whether this was accurate in our system, we performed mammosphere formation assays to propagate stem cells in vitro^29^. We observed that mammospheres formed by the parental cells were regular and compact, whereas the resistant cells formed more disorganized structures (Fig. 2d), which may be associated with their lower E-cadherin expression. Furthermore, T47D-TR cells exhibited a higher mammosphere formation frequency, as shown by ELDA analysis (Fig. 2d), along with increased expression of stemness markers NANOG and OCT4. Additionally, T47D-PR cells showed higher levels of BCRP, a membrane transporter involved in multidrug resistance (Fig. 2e).

To further characterize the resistant phenotype and identify associated acquired mutations, we performed whole exome sequencing (WES) of the T47D variants (Supplementary Table S1). Mutations that were not present in the parental cells were filtered by potential pathogenicity, and mutated genes were then grouped according to their function or involvement in cellular processes based on the Reactome pathway database and bibliographic research. Interestingly, in all resistant variants, the altered genes were primarily associated with epithelial-mesenchymal transition and cell migration (23–26% of the total mutated genes), stemness (13–15%), and regulation of metabolism (10–17%), followed by apoptosis/autophagy (11–15%), and cell cycle regulation (8–10%) (Fig. 2f). Some of the mutated genes that could potentially be associated with resistance to either tamoxifen or palbociclib are shown in the Venn diagram (Fig. 2f, Supplementary Fig. S1f). Further studies are required to validate the role of these genes in the development of resistance. Interestingly, we did not find any new mutations in CDKs, cyclin D1, cyclin E, or hormone receptor genes. It remains to be determined whether copy number variation and epigenetic or post-transcriptional regulation of these genes are involved.

### mTOR inhibition reduces proliferation, migration and mammosphere-forming capacity in the resistant cells

**Figure 3:**
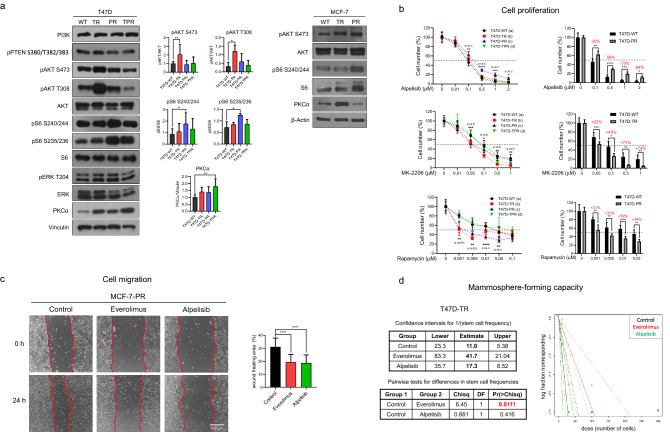
Targeting PI3K/AKT/mTOR pathway in the resistant cells. (**a**) Activation of PI3K/AKT/mTOR pathway. Protein lysates were obtained under basal conditions and analyzed by immunoblot with the indicated antibodies. For T47D variants, bands were quantified by densitometry and relativized to their loading control. *p < 0.05, **p < 0.01, ***p < 0.001. Data represent mean ± SD, one-way ANOVA followed by Dunnett's test (independent replicates n = 3, with 3 experimental replicates in each group). (**b**) Effect of PI3K/AKT/mTOR inhibitors on cell proliferation. Cells were treated with increasing concentrations of alpelisib (PI3Kα inhibitor), MK-2206 (pan-AKT inhibitor) and rapamycin (mTORC1 inhibitor) for 7 days and counted at the end of the experiment. Bar graphs on the right show the percentage (red numbers) of increase (+) or decrease (−) in inhibition of cell proliferation at the different concentrations. *p < 0.05, **p < 0.01, ***p < 0.001, ****p < 0.0001. Data represent mean ± SD at each drug concentration, two-way ANOVA followed by Dunnett's test (independent replicates n = 2, with 4 experimental replicates in each group). (**c**) Effect of PI3K/AKT/mTOR inhibitors on cell migration. Wound healing assays were performed in MCF-7-PR cells. Cells were treated with alpelisib (0.1 µM), everolimus (0.1 µM) or vehicle for 24 h. Representative pictures at the beginning (T0) and at the end (Tf) are shown on the left panel. The wound healing area was quantified as T0-Tf (right). ****p < 0.0001. Data represent mean ± SD, one-way ANOVA followed by Dunnett's test (independent replicates n = 2, with 4 experimental replicates in each group). (**d**) Effect of PI3K/AKT/mTOR inhibitors on mammosphere-forming capacity. Extreme limiting dilution assays were performed in T47D-TR cells. Cells were treated with alpelisib (0.1 µM), everolimus (0.1 µM) or vehicle for 7 days. Left: Sphere formation frequencies were compared using the ELDA web tool. Right: The number of cells seeded per well versus the logarithmic fraction of wells without any detected spheres was plotted. Trend lines represent the estimated active cell frequency, dotted lines show the 95% confidence interval. P-values for significant differences between treatments were calculated using ELDA webtool software (independent replicates n = 2, with 6 experimental replicates in each group). Original blots/gels are presented in Supplementary Material, unprocessed western blots section. Original images are presented in Supplementary Material, unprocessed photomicrographs section.

Previous studies have reported that PI3K/AKT/mTOR and MAPK pathways are involved in the development of resistance to tamoxifen and palbociclib. Therefore, we assessed the expression and activation of proteins associated with these pathways by western blot analysis. Although we did not observe differences in ERK and PTEN phosphorylation or PI3K expression in the resistant variants compared to the parental cells (Fig. 3a), we observed enhanced AKT phosphorylation in the tamoxifen-resistant cells and S6 phosphorylation in the palbociclib-resistant cells (Fig. 3a). Surprisingly, AKT and S6 were not simultaneously overactivated, as expected. Additionally, we found upregulation of PKCα in the tamoxifen-resistant variant MCF-7-TR and in the double-resistant variant T47D-TPR.

We next examined whether the decreased ER expression and AKT/S6 overactivation found in the resistant cells were permanent changes or whether they were induced by the continuous presence of the inhibitors in the culture media. After more than 13 days of drug-free growth, resistant cells still exhibited lower ER expression and increased AKT/S6 phosphorylation, confirming that these changes were not reversible upon drug removal (Supplementary Fig. S2a,b).

Given the increased activation of PI3K/AKT/mTOR signaling observed in the resistant cells, we hypothesized that they would be especially sensitive to the inhibition of this pathway. Therefore, we evaluated the effect of specific inhibitors on cell proliferation. We tested increasing concentrations of alpelisib (PI3Kα inhibitor), MK-2206 (pan-AKT inhibitor), and rapamycin (mTORC1 inhibitor) on each resistant variant and compared their responses to those of the parental cells. Interestingly, alpelisib had a lower antiproliferative effect in the palbociclib-resistant cells, showing up to a 73% reduction in the response. In contrast, MK-2206 was more effective in the tamoxifen-resistant cells, in agreement with their higher AKT phosphorylation levels, with up to a 74% increase in the response. Finally, rapamycin was more effective in both T47D-TR and T47D-PR, with up to 39% increased response in the palbociclib-resistant cells, which exhibited higher phosphorylation of S6, a downstream target of mTOR (Fig. 3b). Remarkably, ten times lower concentrations of rapamycin than alpelisib were already effective in reducing cell proliferation.

Next, we assessed the influence of PI3K/AKT/mTOR inhibition on cell migration and stemness, two processes that were altered in the resistant cells. Treatment with everolimus (rapamycin analog) and alpelisib reduced the migration of the palbociclib-resistant MCF-7 cells (Fig. 3c). However, only everolimus successfully diminished the mammosphere-forming capacity in the tamoxifen-resistant T47D cells (Fig. 3d). We performed these two analyses in the resistant cell variants that exhibit the highest migratory or mammosphere-forming capacity compared to the parental cells (Fig. 2c,d).

### Combined inhibition of mTOR and CDK4/6 shows an enhanced antitumor effect in the resistant cells

**Figure 4:**
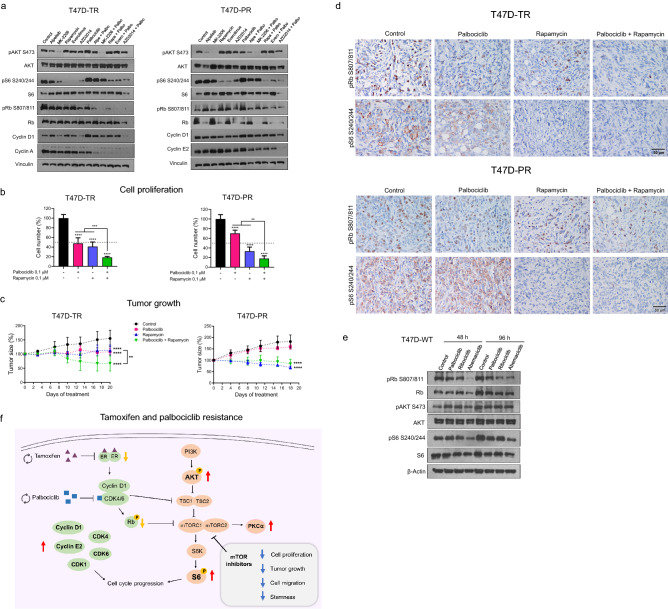
Combined inhibition of mTOR and CDK4/6 to overcome resistance. (**a**) Effect of palbociclib in combination with PI3K/AKT/mTOR inhibitors on cell cycle proteins and PI3K/AKT/mTOR activation. T47D-TR and T47D-PR cells were treated with alpelisib (0.1 µM), MK-2206 (0.1 µM), rapamycin (0.1 µM), everolimus (0.1 µM), AZD2014 (0.1 µM) and their combinations with palbociclib (0.1 µM) for 30 h. Protein lysates were analyzed by immunoblot with the indicated antibodies. (**b**) Effect of palbociclib in combination with rapamycin on cell proliferation. T47D-TR and T47D-PR cells were treated with palbociclib (0.1 μM), rapamycin (0.1 µM) and the combination for 7 days. **p < 0.01, ***p < 0.001, ****p < 0.0001. Data represent mean ± SD, one-way ANOVA followed by Tukey's test (independent replicates n = 3, with 4 experimental replicates in each group). (**c**) Effect of palbociclib in combination with rapamycin on tumor growth. 8 × 10^6^ T47D-TR or T47D-PR cells were inoculated subcutaneously in the lateral flank of NSG mice. Tumor sizes were relativized to their size before starting treatment. Tumors were treated with palbociclib (25 mg/kg 5 times per week, subcutaneously), rapamycin (17.5 mg/kg 2 times per week, by intraperitoneal injection) or the combination for 18–20 days. **p < 0.01, ****p < 0.0001. Data represent mean ± SD, two-way ANOVA followed by Tukey’s test (independent replicates n = 2, with 3 to 6 experimental replicates in each arm of treatment). Representative curve of two in each variant. (**d**) Effect of palbociclib in combination with rapamycin on pRb and pS6 levels in tumors. Immunohistochemistry for pRb Ser807/811 and pS6 Ser240/244 was performed on tumors treated with palbociclib, rapamycin, and the combination. Representative images of stained tumor sections. (**e**) Effect of different CDK4/6 inhibitors on PI3K/AKT/mTOR activation. T47D-WT cells were treated with palbociclib (0.1 µM), ribociclib (0.1 µM), abemaciclib (0.1 µM) or vehicle for 48 or 96 h. Protein lysates were analyzed by immunoblot with the indicated antibodies. (**f**) Summary of the results found in T47D and MCF-7 resistant cell variants. Red/yellow arrows represent upregulation/downregulation of phosphorylation or protein expression. Blue arrows represent downregulation of cellular processes. Original blots/gels are presented in Supplementary Material, unprocessed western blots section.

We next evaluated the benefits of incorporating specific PI3K/AKT/mTOR inhibitors into palbociclib therapy for the resistant variants. First, the effect of the inhibitors on target proteins was verified by western blotting (Fig. 4a). As expected, alpelisib and MK-2206 decreased AKT phosphorylation and, to a lesser extent, S6 phosphorylation in the tamoxifen-resistant cells. However, alpelisib failed to inhibit S6 phosphorylation in the palbociclib-resistant cells, likely because of its higher basal pS6 levels, which could account for the observed lower antiproliferative effect of alpelisib in these cells (Fig. 3b). As expected, all mTOR inhibitors (including AZD2014, a dual mTORC1/2 inhibitor) efficiently inhibited S6 phosphorylation in all the resistant variants (Fig. 4a, Supplementary Fig. S2c). Furthermore, palbociclib inhibited Rb phosphorylation only in tamoxifen-resistant cells but increased the expression of cyclin D1 in both T47D-TR and T47D-PR cells. The combination of mTOR inhibitors with palbociclib deepened the decrease of cyclin D1, cyclin E2, and cyclin A expression as well as pRb and pS6 inhibition (Fig. 4a, Supplementary Fig. S2c). We subsequently found that, in all T47D resistant variants, combined treatment with rapamycin and palbociclib reduced cell proliferation to a greater extent than the treatment with each inhibitor alone (Fig. 4b, Supplementary Fig. S2d).

Regarding tumor growth, the combined treatment was more effective than each drug alone, promoting tumor regression in the tamoxifen-resistant variant. In the palbociclib-resistant variant, rapamycin as monotherapy was able to induce tumor regression, and the combination with palbociclib did not induce a greater effect (Fig. 4c). It is relevant to note that the resistant cells/xenografts were able to grow even in presence of drugs, although at a lower rate than the parental cell lines. Tumor immunostaining revealed that both palbociclib and rapamycin alone diminished pRb and pS6 expression in tamoxifen-resistant tumors, whereas the combined treatment improved the inhibitory effect (Fig. 4d). In palbociclib-resistant tumors, treatment with either palbociclib or rapamycin was insufficient to inhibit pRb and pS6, whereas their combination decreased the phosphorylation of both proteins.

Some studies have shown that the cyclin D1/CDK4/6 complex can activate the PI3K/AKT/mTOR pathway through inhibition of TSC2, a negative regulator of mTORC1^21,22^. Since we found that inhibition of the cyclin D1/CDK4/6/Rb axis with palbociclib did not cross-regulate AKT or S6 phosphorylation in the palbociclib- and double-resistant models (Fig. 4a, Supplementary Fig. S2c), we also evaluated the cross-effect of CDK4/6 inhibitors in sensitive T47D and MCF-7 wild-type cells. In this context, abemaciclib was most effective in reducing both Rb and S6 phosphorylation, although AKT remained active (Fig. 4e, Supplementary Fig. S2e).

### Effect of PI3K/AKT/mTOR inhibition on proliferation of tamoxifen- and palbociclib-resistant PDCs

**Figure 5:**
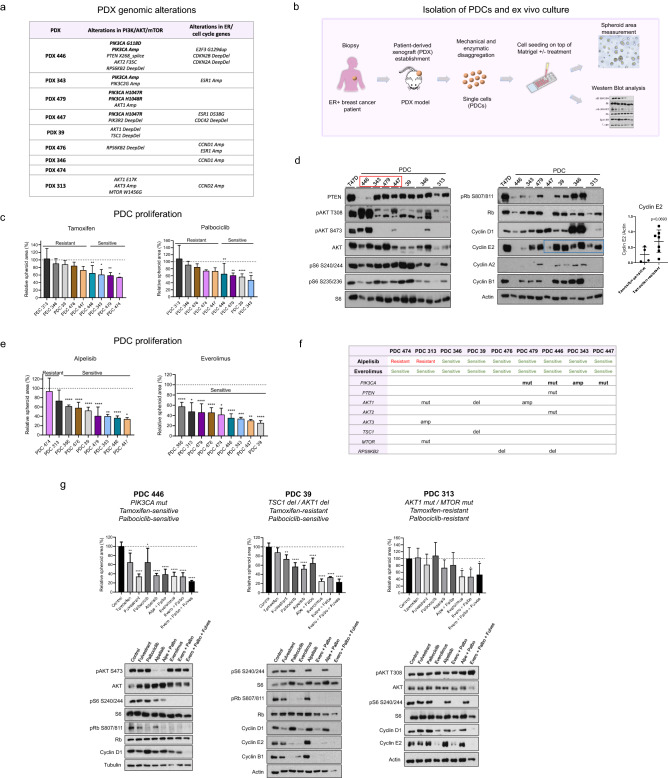
Targeting PI3K/AKT/mTOR pathway in patient-derived cells. (**a**) Genomic alterations in PI3K/AKT/mTOR pathway, ER and cell cycle genes in PDXs. (**b**) Isolation of PDCs and ex vivo culture. (**c**) Sensitivity to tamoxifen and palbociclib. PDCs were treated with tamoxifen (0.1 μM), palbociclib (0.5 μM) or vehicle for 7 days. Waterfall plots represent the response of each PDC to treatment. The average area of treated spheres was relativized to the area of the control spheres (vehicle). PDCs were grouped as sensitive if significant differences in sphere area compared to their respective control were found; otherwise, they were classified as resistant. *p < 0.05, **p < 0.01, ****p < 0.0001. Data represent mean ± SD, one-way ANOVA followed by Tukey's test (independent replicates n = 2, with 2 experimental replicates in each group). (**d**) Activation of PI3K/AKT/mTOR pathway and expression of cell cycle proteins. Protein lysates from PDCs were analyzed by immunoblot with the indicated antibodies. PDCs harboring alterations in *PIK3CA* (red box), did not necessarily exhibit higher AKT or S6 phosphorylation levels. Tamoxifen-resistant PDCs showed an upward trend in cyclin E2 expression (blue box). Bands were quantified by densitometry and relativized to their loading control (bar graph). Data represent mean ± SD, two-sided Student’s t-test. (**e**) Sensitivity to alpelisib and everolimus. PDCs were treated with alpelisib (1 μM), everolimus (0.1 μM) or vehicle for 7 days. Waterfall plots represent the response of each PDC to treatment. The average area of treated spheres was relativized to the area of the control spheres (vehicle). PDCs were grouped as sensitive if significant differences in sphere area compared to their respective control were found; otherwise, they were classified as resistant. *p < 0.05, **p < 0.01, ***p < 0.001, ****p < 0.0001. Data represent mean ± SD, one-way ANOVA followed by Tukey's test (independent replicates n = 2, with 2 experimental replicates in each group). (**f**) Genomic alterations in PI3K/AKT/mTOR pathway. Alpelisib-resistant PDCs (474 and 313) presented *PIK3CA-wt*, while four out of seven  alpelisib-sensitive PDCs harbored alterations in *PIK3CA*. All PDCs were sensitive to everolimus regardless of the presence of alterations in the PI3K/AKT/mTOR pathway. (**g**) Combined treatment with palbociclib, alpelisib/everolimus and fulvestrant. PDCs were treated with tamoxifen (0.1 µM), fulvestrant (0.1 µM), palbociclib (0.5 µM), alpelisib (1 µM), everolimus (0.1 µM) and their combinations for 7 days (sensitivity analysis, top panels) or 30 h (western blot, bottom panels). *p < 0.05, **p < 0.01, ***p < 0.001, ****p < 0.0001. Data represent mean ± SD, one-way ANOVA followed by Tukey's test (independent replicates n = 2, with 2 experimental replicates in each group). Original blots/gels are presented in Supplementary Material, unprocessed western blots section.

To assess whether the results found in cell lines (summarized in Fig. 4f) could be validated in models that better reflect the complexity and heterogeneity of patient tumors, we used nine PDXs established from ER + breast tumors. Genomic alterations in the PDXs have been previously analyzed using the MSK-IMPACT panel^30^. Some of the relevant alterations associated with the PI3K/AKT/mTOR and cyclin D1/CDK4/6/Rb pathways are shown in Fig. 5a.

Cells were isolated from PDXs (PDCs), as described in “Materials and Methods” (Fig. 5b). We performed ex vivo assays using PDCs to evaluate their response to ER, PI3K/AKT/mTOR, and CDK4/6 inhibitors as well as the expression and activation of proteins associated with these pathways. The sensitivity of each PDC to tamoxifen and palbociclib is shown in Fig. 5c.

The expression of PTEN remained mostly constant among the different PDCs, regardless of their sensitivity to tamoxifen and palbociclib (Fig. 5d). When analyzing PI3K/AKT/mTOR activation, we observed that alterations in the *PIK3CA* gene did not always correlate with greater AKT or S6 phosphorylation (Fig. 5d, red box). Furthermore, pS6 did not always correlate with pAKT levels. Regarding the cell cycle, we did not find an association between palbociclib resistance and changes in Rb expression. However, tamoxifen-resistant PDCs exhibited an upward trend in cyclin E2 expression (Fig. 5d, blue box), similar to that observed in the tamoxifen- and double-resistant cells (Fig. 1f).

Further assessment of the PDCs response to PI3K and mTOR inhibition revealed that only two of them, PDC474 and PDC313, were resistant to alpelisib, while all of them were sensitive to everolimus (Fig. 5e). Interestingly, both alpelisib-resistant PDCs lacked *PIK3CA* mutations, although PDC313 showed alterations in *AKT1*, *AKT3* and *MTOR*. Among the seven alpelisib-sensitive PDCs, four (PDC479, PDC446, PDC343, and PDC447) presented either mutations or amplifications in *PIK3CA,* whereas two (PDC39 and PDC476) exhibited other pathway-associated alterations. Moreover, all PDCs were sensitive to everolimus, regardless of the presence of alterations in *PIK3CA* or downstream genes (Fig. 5f).

Finally, we evaluated the PDCs response to combined inhibition of PI3K/AKT/mTOR and CDK4/6. Additionally, since these inhibitors are typically used in the clinic in combination with endocrine therapy, we tested the effect of incorporating the ER degrader fulvestrant. We analyzed three PDCs with different genomic alterations and responses to tamoxifen and palbociclib ex vivo. Treatment with PI3K or mTOR inhibitors as monotherapy significantly reduced cell proliferation, although the incorporation of palbociclib and fulvestrant did not improve the response (Fig. 5g). When we analyzed the effect of these inhibitors on PI3K/AKT/mTOR activation and cell cycle proteins, we found that palbociclib did not affect AKT/S6 phosphorylation but increased cyclin D1 levels in all models. Alpelisib decreased pRb, pAKT, and pS6 levels only in PDC446, which harbors *PIK3CA* mutations. In contrast, everolimus alone or in combination with palbociclib successfully reduced pS6, pRb, cyclin D1, cyclin E2, and cyclin B1 levels in all the models, regardless of their sensitivity to tamoxifen/palbociclib and the presence of genomic alterations in the PI3K/AKT/mTOR pathway. Finally, the addition of fulvestrant resulted in more effective inhibition of cyclin D1 expression in all cases (Fig. 5g).

## Discussion

In this study, we aimed to elucidate the mechanisms involved in resistance to current therapies used in ER + breast cancer by characterizing preclinical models of first- and second-line resistance to tamoxifen and palbociclib. Since palbociclib is generally indicated for patients who relapse after hormonal treatment, we also developed a double-resistant model that better represents this therapeutic sequence.

A significant decrease in ER and PGR expression was observed in the resistant cells, affecting their response to ER and CDK4/6 inhibitors. Tamoxifen-resistant cells exhibited cross-resistance with the ER inhibitor fulvestrant, while showing a significantly lower response to CDK4/6 inhibitors than the parental cells. Palbociclib-resistant cells also displayed resistance to other CDK4/6 inhibitors such as ribociclib and abemaciclib. Interestingly, their response to ER inhibitors was also significantly reduced, which was consistent with their lower ER levels. Similar results were shown by Kettner et al.^31^ although here we incorporated the double-resistant variant. These data support the idea that ER and CDK4/6 inhibitors share some resistance mechanisms.

Despite their lower proliferation rates, the resistant cells displayed increased expression of cell cycle proteins. In particular, cyclin E2 was found to be increased in both resistant cell lines and tamoxifen-resistant PDCs. Overexpression of cyclin E was previously reported as a mechanism to evade CDK4/6 inhibition because it can activate CDK2 and Rb phosphorylation to re-enter the cell cycle, as observed in preclinical studies^18,19,32,33^ and retrospective analyses of tumor tissues^34,35^. We hypothesize that the reduced proliferation rates in the resistant cells may act as a mechanism of survival, since the inhibitors with which they were selected affect directly cell cycle progression.

Furthermore, the resistant variants displayed overactivation of the PI3K/AKT/mTOR pathway at AKT or S6 levels. WES analysis confirmed that all T47D variants harbored *PIK3CA*-activating mutation H1047R, as previously described for this cell line. However, the resistant cells did not acquire new mutations in *PIK3CA* or other relevant mediators of the PI3K/AKT/mTOR pathway, which could account for the increased phosphorylation of AKT/S6 observed in these cells. It remains to be determined whether other types of genetic, epigenetic, or post-transcriptional modifications are involved in the overactivation of this pathway. In particular, analysis of copy number variation, ubiquitination, sumoylation and microRNA regulation of pathway effectors in the resistant cells are under investigation. Given the fact that resistant cells displayed AKT and S6 hyperactivation, we hypothesized that they would be particularly sensitive to downstream inhibitors of the pathway. Consistently, the mTORC1 inhibitor rapamycin was more effective than the PI3Kα inhibitor alpelisib in reducing proliferation in tamoxifen- and palbociclib-resistant cells, even at low concentrations.

We also discovered that resistance to tamoxifen and palbociclib co-occurs with a more disorganized cell phenotype and loss of cell–cell adhesion, which could influence the migratory and invasive capacity of the cells. Interestingly, we found that both PI3K and mTOR inhibitors reduced cell migration in the palbociclib-resistant model.

Previous studies have reported an association between the increased expression of SOX2 and other stemness markers and resistance to tamoxifen in cell lines and human tumors^36,37^. Furthermore, downregulation of luminal/epithelial differentiation markers and upregulation of basal/mesenchymal invasive markers was reported in MCF7 TamR cells^38^. In this study, we found stem cell enrichment in the tamoxifen-resistant cells, thereby constituting a potential way to escape treatment. We subsequently examined the effects of PI3K/AKT/mTOR inhibitors on stem cell populations. Similar to what was previously reported in a palbociclib-resistant context^39^, everolimus but not alpelisib, interfered with the mammosphere-forming capacity of tamoxifen-resistant cells. However, the role of mTOR in the regulation of stemness is not fully understood, and additional studies are required to confirm these findings, considering that mTOR inhibition may have a long-term beneficial effect on reducing tumor recurrence.

Currently, there are different clinical trials evaluating the use of PI3K/AKT/mTOR inhibitors in combination with endocrine therapy for the treatment of patients with ER + advanced or metastatic breast cancer who relapse after a first- or second-line treatment. Examples include PI3K (NCT03056755), AKT (NCT01277757), and mTOR (NCT02871791, NCT02732119, NCT02216786, NCT01805271) inhibitors. These treatments are also being evaluated in clinical trials for other types of cancers^40,41^.

Although the presence of mutations in the *PIK3CA* gene determines the administration of alpelisib to patients who relapse after endocrine therapy, the prognostic and predictive values of these mutations are still being questioned. Previous studies have indicated that *PIK3CA* mutations do not always correlate with higher pathway activation^13,42–44^ or do not necessarily predict sensitivity to PI3K inhibition^45^, possibly due to the presence of alterations in other components of this pathway or in other pathways that can mediate resistance. Moreover, AKT phosphorylation does not always correlate with phosphorylation of the downstream protein S6, as we observed in both resistant cells and PDCs. Two possible explanations for this phenomenon are that S6 can also be regulated by other signaling pathways, such as MAPK/ERK and PKC^46^ or that S6K can negatively regulate IRS-1, consequently decreasing AKT activation^47^. Our findings suggest that analyzing PI3K/AKT/mTOR pathway activation beyond *PIK3CA* mutation status is crucial for the proper selection of patients that could potentially receive treatment with specific inhibitors, maximizing therapeutic response. As S6 is a downstream target of mTOR, immunohistochemical detection of S6 phosphorylation could aid in identifying patients who would benefit from a therapy that targets mTOR. Additional preclinical and clinical studies are required to investigate whether high pS6 levels indicate a weak or null therapeutic response to PI3K inhibitors.

The PI3K/AKT/mTOR and cyclin D1/CDK4/6/Rb pathways cross-regulate each other at several nodes^5^. An interesting finding from our study was that palbociclib, while inducing an increase in cyclin D1 levels, did not efficiently cross-regulate PI3K/AKT/mTOR activation at AKT or S6 levels, suggesting that mTOR remains active despite palbociclib treatment**.** This finding agrees with some previous studies^18–20^ but contrasts with others that state that the active cyclin D1/CDK4/6 complex activates the PI3K/AKT/mTOR pathway^21,22^. Furthermore, in concordance with previous studies^48^, we found that abemaciclib was the CDK4/6 inhibitor with the highest efficiency in inhibiting both Rb and S6 phosphorylation. Additional studies are needed to investigate whether switching to other CDK4/6 inhibitors after the appearance of resistance would be beneficial.

The combination of PI3K/AKT/mTOR and CDK4/6 inhibitors has been studied in preclinical models^18,20,49^ and is still under research in clinical trials in patients with advanced breast cancer (NCT02389842), lung cancer and other cancers (NCT03065062). Combination therapy with CDK4/6 and mTOR inhibitors could also enable the use of lower doses, thus diminishing adverse effects and toxicity in patients, as neutropenia, leukopenia, thrombocytopenia, anemia, fatigue or diarrhea.

Here, we showed that the use of mTOR inhibitors was effective as monotherapy in the case of PDCs. Furthermore, mTOR inhibitors in combination with CDK4/6 inhibitors resulted in a greater reduction of cell proliferation and tumor growth. We speculate that this reduction is mainly due more to cell cycle arrest. This information would be relevant in studying strategies to prevent/solve therapy resistance. So far, we found that both in PDCs and cell lines, the combination limited the activation of cyclin D1/CDK4/6/Rb and PI3K/AKT/mTOR pathways simultaneously in the context of resistance to either tamoxifen or palbociclib, irrespective of *PIK3CA* mutation status. This combination would be necessary to suppress the reactivation of the PI3K/AKT/mTOR pathway downstream of mTOR more efficiently and potentially prevent or delay the emergence of further resistance to CDK4/6 inhibitors.

## Materials and methods

### Cell lines

T47D (RRID:CVCL_0553) and MCF-7 (RRID:CVCL_0031) cell lines were purchased from ATCC and cultured in phenol red-free DMEM/F12 medium (GIBCO, Waltham, Massachusetts, USA) containing 10% fetal bovine serum (FBS) and 1 nM insulin. Tamoxifen- (TR) and palbociclib-resistant (PR) variants were generated by long-term exposure of wild-type cells (WT) to increasing concentrations of either 4-hydroxytamoxifen or palbociclib for 12 months up to 1 µM. Double-resistant cells (TPR) were obtained by treating tamoxifen-resistant cells with palbociclib for 12 months. Inhibitors were removed 24–48 h before each experiment to compare with culture conditions of wild type cells in the absence of drugs. All experiments were performed using mycoplasma-free cells. All cell lines have been authenticated using STR profiling within the last year. The majority of experiments were performed in the T47D variants, which were generated first. The MCF7 variants were generated in a later stage to confirm the most relevant findings obtained in T47D.

### In vitro experiments

#### Proliferation assays

To compare cell proliferation rates, cells grown in DMEM/F12 with 10% FBS were counted every 2–3 days using a Countess II cell counter (Thermo Fisher Scientific, Waltham, Massachusetts, USA). For inhibitor response analysis, cells grown in DMEM/F12 with 7% FBS were treated with either vehicle or inhibitors and counted after 7 days of treatment when we observe better differences between treatments. In this case, we considered end-time cell count.

#### Wound healing assays

To evaluate cell migration, cultures at 95% confluence were scratched with a pipette tip and incubated in DMEM/F12 with 2% FBS and either vehicle or inhibitors for 24 h. Images were taken along the scratch at the beginning (T0) and after 24 h (Tf) using an Olympus CKX41 microscope. The wound healing area was quantified as T0-Tf using ImageJ software. Migration was evaluated only in MCF-7 cells, since T47D cells have a verly low migratory capacity.

#### Transwell assays

7.5 × 10^4^ cells in 200 µl of serum-free medium were seeded onto 8 μm-pore inserts in 24-well plates containing 500 μl of DMEM/F12 with 10% FBS. After 24 h, cells were fixed with methanol and stained with crystal violet 0.1%. Images were acquired using a Nikon Eclipse E800 microscope, and cells that passed through the inserts were quantified using the ImageJ software.

#### Cell cycle analysis

Cells were starved in serum-free DMEM/F12 and then incubated for 24 h in a medium with 10% FBS, followed by trypsinization, ethanol 70% fixation and incubation with 20 µg/ml propidium iodide (Sigma-Aldrich, St Louis, Missouri, USA) and 100 µg/ml RNase A (Invitrogen, Waltham, Massachusetts, USA) for 1 h. Samples were analyzed using a BD FACS Canto II (Becton Dickinson, Franklin Lakes, New Jersey, USA) flow cytometer and data were processed using FlowJo10 software. For details about the protocol and analysis, see Supplementary Materials and Methods.

#### Mammospheres and extreme limiting dilution assays (ELDA)

To assess mammosphere morphology, single-cell suspensions were seeded in 6-well plates (3000 cells/ml) treated with poly-(2-hydroxyethyl methacrylate) (poly-HEMA) to prevent cell adhesion to the plate. Cells were cultured in serum-free DMEM/F12 medium supplemented with 20 ng/ml EGF, 20 ng/ml bFGF, and B-27 supplement 1X (Gibco). After seven days, the formed spheres were photographed using an Olympus CKX41 microscope. The mammosphere assay was only attempted in T47D cell variants.

The mammosphere-forming capacity at baseline and after treatment was quantified by ELDA^50^. Briefly, single-cell suspensions were plated in poly-HEMA-treated 96-well plates at 300, 100, 30, and 10 cells/well. After 7 days of incubation with either vehicle or inhibitors, wells with at least one mammosphere were counted for each plating density, and stem cell frequency was calculated using the ELDA analysis software (https://bioinf.wehi.edu.au/software/elda/).

#### Quantitative real-time PCR (qRT-PCR)

For details about the protocol see Supplementary Materials and Methods.

#### Western blot

To analyze the basal activation of signaling pathways, cells were incubated in serum-free DMEM/F12 for 24 h. To evaluate their response to inhibitors, cells were incubated in a medium with 7% FBS and treated with either vehicle or inhibitors for 30 h. After incubation, cells were lysed with RIPA buffer supplemented with a protease inhibitor cocktail (Sigma-Aldrich), and phosphatase inhibitors β-glycerophosphate (Chem Cruz, Dallas, Texas, USA) and sodium fluoride (Cicarelli, Santa Fe, Argentina). 25 μg of total protein from each sample were resolved by SDS-PAGE and transferred to nitrocellulose membranes, which were blocked with 5% non-fat dry milk/TBS-Tween and incubated with primary antibodies at 4 °C overnight, followed by incubation with HRP-conjugated secondary antibodies (Vector Laboratories, Burlingame, California, USA) for 1 h at room temperature. Immunoreactive bands were visualized by enhanced chemiluminescence and quantified using the ImageJ software. Quantitative comparisons between samples were made in the same gel. Once relativized to the control, the statistics were made between gels (independent experiments). All blots were cut prior to hybridization with antibodies to use different antibodies on the same membrane.

#### Immunofluorescence

Cells grown on glass coverslips were fixed with either 70% ethanol or 4% paraformaldehyde, permeabilized with 0.25% Triton X-100 (only for paraformaldehyde fixation), blocked with 10% FBS, and incubated with primary antibodies at 4 °C overnight. Cells were then incubated with FITC-conjugated secondary antibodies (Vector Laboratories) for 1 h at room temperature. Nuclei were counterstained with 4′,6-diamino-2-phenylindole (DAPI). For 3D growth assays, cells were seeded onto 8-well chamber slides coated with reduced growth factor basement membrane matrix Geltrex (Thermo Fisher Scientific) and cultured for 48 h in DMEM/F12 with 10% FBS, followed by fixation with 4% paraformaldehyde/20% sucrose, and staining with phalloidin-TRITC (Sigma). Nuclei were counterstained with DAPI. Images were obtained with an Olympus DSU IX83 confocal microscope using CellSens Dimensions software.

#### PDCs ex vivo culture

Patient-derived cells (PDCs) were isolated from PDXs through mechanical and enzymatic disaggregation following the protocol described by Bruna et al.^51^. For details about the protocol, see Supplementary Materials and Methods. Details regarding the maintenance of mice and PDXs can be found in Gris-Oliver et al.^52^.

For western blotting, 1 × 10^6^ cells/ml were seeded in 6-well plates coated with Matrigel growth factor reduced (GFR) basement membrane matrix (Corning, New York, USA) and incubated for 48 h. Cells were then treated for 30 h with either vehicle or inhibitors. To evaluate drug sensitivity, 2 × 10^5^ cells/ml were seeded onto 8-well chamber slides coated with Matrigel and incubated for 48 h. PDCs were then treated with either vehicle or inhibitors for 7 days and photographed on day 7. The media and treatments were refreshed every 2–3 days. The sphere size was assessed using ImageJ software, and the average size for each condition was normalized to that of the controls.

#### Whole exome sequencing (WES)

For details about the protocol see Supplementary Materials and Methods.

### Ethics statement

Animal care and manipulation were performed in accordance with international guidelines and regulations of the National Institute of Health. All studies were performed according to protocols approved by the IBYME-IACUC Ethical Committee (Protocol 027/2017). Studies and analysis involving animals follow the recommendations in the ARRIVE guidelines.

### Collection of tumor samples and establishment of patient-derived xenografts

Fresh tumor samples from patients with breast cancer were collected following the Institutional Research Board from Comité de Etica de Investigación con Medicamentos y Comisión Proyectos de Investigación del Hospital Universitari Vall D’Hebron approved the protocol PR(AG)20-2019 and the associated written informed consent. The study was compliant with the declaration of Helsinki. We declare that no tissues were procured from prisoners. For details about the protocol see Supplementary Materials and Methods.

### In vivo experiments

#### Animals

Two-month-old female NOD/LySz-scid/IL-2Rgamma null (NSG) mice purchased from The Jackson Laboratory (Bar Harbor, Maine, USA) and bred at the Animal Facility of Instituto de Biología y Medicina Experimental (IBYME) were used. All animals were fed ad libitum and kept in an air-conditioned room at 20 ± 2 °C with a 12-h light/dark cycle period.

At the end of the experiments, animals were euthanized using carbon dioxide chamber. For details about an animal study design see Supplementary Materials and Methods.

#### Xenografts and treatments

Silastic pellets containing 17β-estradiol (0.25 mg) were subcutaneously implanted into NSG mice. The following day, 8 × 10^6^ cells suspended in DMEM/F12 and Geltrex at a 2:1 ratio were subcutaneously injected into the lateral flank of each mouse, adjacent to the mammary gland. Once tumors were palpable, their size was measured with a Vernier caliper every 3–4 days. To compare the tumor growth rates, curves were plotted for tumor size (mm^2^) over time. Approximately 4 weeks after cell injection, mice bearing 30 mm^2^ tumors were randomized to treatment with vehicle (control), tamoxifen citrate (5 mg/kg five times per week, subcutaneously), palbociclib isethionate (25 mg/kg five times per week, subcutaneously), rapamycin (17.5 mg/kg twice per week, by intraperitoneal injection), or the combination for 2–3 weeks. Animal weight and tumor size were measured twice weekly. To analyze treatment responses, the relative tumor growth (%) was plotted starting on the first day of treatment. After treatment, the tumors, lungs, liver, and kidneys were fixed in 4% formalin, followed by paraffin embedding for immunohistochemistry or toxicity analysis. Before fixation, tumors were harvested 24 h after the last drug dose. General health, body weight, and liver and kidney histology revealed no toxicity after treatment.

#### Immunohistochemistry

For details about the protocol see Supplementary Materials and Methods.

#### Statistical analyses

In the figure legends, “n” represents the number of independent biological replicates, the technical replicates in each group are also indicated.

In vitro and in vivo experiments were performed at least twice. Statistical analyses were performed using GraphPad Prism™ software 8.0. One-way ANOVA followed by Tukey’s or Dunnett’s post-test was used to compare the means of multiple experimental groups. A two-sided Student’s t-test was used to compare the means of two different groups. Differences among cell lines and tumor growth curves were analyzed using two-way ANOVA, followed by Tukey’s multiple comparison test. In all graphs, the mean ± standard deviation SD values are shown. Normality was tested in GraphPad prior to ANOVA using Shapiro–Wilk or Kolmogorov–Smirnov tests.

## Supplementary Information

Supplementary Information 1.

Supplementary Information 2.

Supplementary Information 3.

## Data Availability

The WES data generated in this study are available in NCBI under accession BioProject number PRJNA853765 at https://www.ncbi.nlm.nih.gov/bioproject/PRJNA853765. Other data that support the findings of our study are available from the corresponding author upon request.

**Table:** 

Cell lines	Bioproject	Sequencing data
T47D tamoxifen and palbociclib resistant	PRJNA853765	SRR19976687 SRR19976688
T47D palbociclib resistant	PRJNA853765	SRR19976689 SRR19976690
T47D tamoxifen resistant	PRJNA853765	SRR19976691 SRR19976692
T47D wild type	PRJNA853765	SRR19976693 SRR19976694 SRR19976687 SRR19976688 SRR19976689 SRR19976690 SRR19976691 SRR19976692 SRR19976693 SRR19976694

## Abbreviations

DAPI: 4′,6-Diamidino-2-phenylindole
ELDA: Extreme limiting dilution assay
ER: Estrogen receptor
FBS: Fetal bovine serum
NSG: NOD/LtSz-scid/IL-2Rgamma null
PDCs: Patient-derived cells
PDXs: Patient-derived xenografts
PFA: Paraformaldehyde
poly-HEMA: Poly-(2-hydroxyethyl methacrylate)
PR: Palbociclib-resistant
qRT-PCR: Quantitative real-time PCR
TPR: Tamoxifen + palbociclib resistant
TR: Tamoxifen-resistant
WES: Whole exome sequencing
