# Supplementary material for: Targeting mTOR to overcome resistance to hormone and CDK4/6 inhibitors in ER-positive breast cancer models
Source: Sci Rep. 2023 Feb 15;13:2710. doi: 10.1038/s41598-023-29425-y (PMC9932145; doi:10.1038/s41598-023-29425-y)
Supplement: Supplementary file 1 — Supplementary Information 1. [file 41598_2023_29425_MOESM1_ESM.pdf]

Targeting mTOR to overcome resistance to hormone and CDK4/6 inhibitors in ER-positive breast cancer models.

María Jimena Rodriguez, María Cecilia Perrone, Marina Riggio, Marta Palafox, Valeria Salinas, Andrés Elia, Natali Daiana Salgueiro, Andrea Eugenia Werbach, María Paula Marks, Marcelo A. Kauffman, Luciano Vellón, Violeta Serra, Virginia Novaro.

SUPPLEMENTARY FIGURES AND LEGENDS

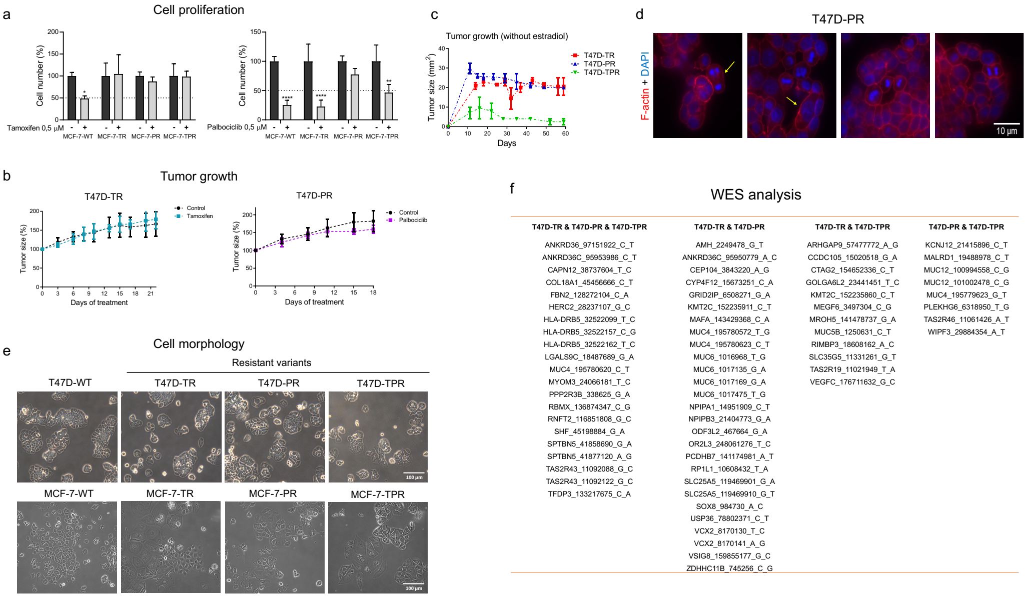

Supplementary Figure S1. Characterization of the resistant cells.

(a) Effect of tamoxifen and palbociclib on cell proliferation. MCF7 cell variants were treated with 4-hydroxytamoxifen (0.5 µM), palbociclib (0.5 µM) or vehicle for 7 days and counted at the end of the experiment. *p<0.05, **p<0.01, ***p<0.001 compared to their respective control. Data represent mean ± SD, two-way ANOVA followed by Tukey 's test (independent replicates n=3, with 4 experimental replicates in each group).

(b) Effect of tamoxifen and palbociclib on tumor growth. 8x10^6 T47D-TR or T47D-PR cells were inoculated subcutaneously in the lateral flank of NSG mice. Tumor sizes were relativized to their size before starting treatment. T47D-TR tumors were treated with tamoxifen (5 mg/kg 5 times per week, subcutaneously) and T47D-PR tumors were

treated with palbociclib (25 mg/kg 5 times per week, subcutaneously). T47D-TR and T47D-PR xenografts did not respond to tamoxifen and palbociclib respectively. Data represent mean ± SD. P-values for significant differences between treatments was calculated using two-way ANOVA followed by Tukey's multiple comparison test (independent replicates n=2, with 3 experimental replicates in each group). Representative curve of two in each variant.

(c) Tumor growth curves without estradiol supplementation. $8x10^6$ cells of each variant were injected subcutaneously in the lateral flank of NSG mice. Without estradiol supplementation, the resistant cells formed small tumors, unable to grow any further. Data represent mean ± SD. (independent replicates n=2, with 3 experimental replicates in each group).

(d) Mitotic cells in T47D-PR variant. Representative confocal microscopy images. Cells were stained for F-actin with phalloidin (red), and nuclei were counterstained with DAPI. Although most mitosis were normal, some tripolar mitosis and giant cells with irregular nuclei were found (yellow arrows).

(e) T47D and MCF-7 2D cultures. The resistant cells exhibited heterogeneity in size and morphology.

(f) Pathogenic mutations in T47D resistant cells. Mutations that were not present in the parental cells were filtered by potential pathogenicity using REVEL, SIFT, Polyphen2 and CLNSIG predictors. Table showing the pathogenic mutations shared among the resistant variants.

Original images are presented in Supplementary Material, unprocessed photomicrographs section.

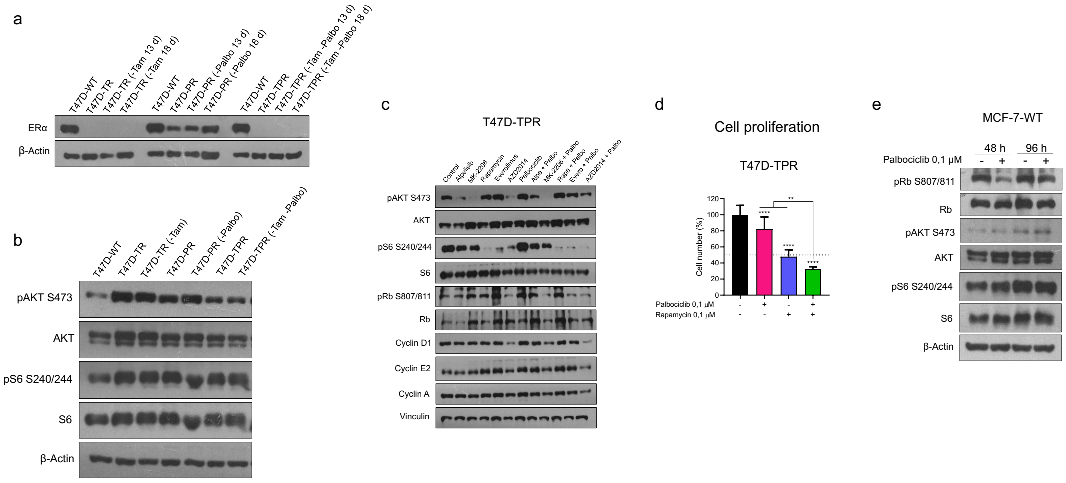

Supplementary Figure S2. Regulation of PI3K/AKT/mTOR pathway in wild-type and resistant cells.

(a,b) ER expression and PI3K/AKT/mTOR activation upon removal of selection drugs. Cells were cultured in media without tamoxifen or palbociclib for 13 and 18 days. Protein lysates were analyzed by immunoblot with the indicated antibodies. The resistant cells still exhibited lower ER expression (A) and increased AKT/S6 phosphorylation (B) after culturing in drug-free conditions, confirming that these changes are not reversible.

(c) Effect of palbociclib in combination with PI3K/AKT/mTOR inhibitors on cell cycle proteins and PI3K/AKT/mTOR activation. T47D-TPR cells were treated with alpelisib (0.1 µM), MK-2206 (0.1 µM), rapamycin (0.1 µM), everolimus (0.1 µM), AZD2014 (0.1 µM) and their combinations with palbociclib (0.1 µM) for 30 hours. Protein lysates were analyzed by immunoblot with the indicated antibodies. Palbociclib was not able to reduce Rb phosphorylation, while its combination with PI3K/AKT/mTOR inhibitors decreased pS6, pRb, cyclin D1, cyclin E2 and cyclin A to a greater extent.

(d) Effect of palbociclib in combination with rapamycin on cell proliferation. T47D-TPR cells were treated with palbociclib (0.1 µM), rapamycin (0.1 µM) and the combination for 7 days. The combined treatment showed a greater inhibitory effect compared to the individual drugs. **p<0.01, ****p<0.0001. Data represent mean ± SD. P-values for significant differences between treatments was calculated using one-way ANOVA,

followed by Tukey's multiple comparison test (independent replicates n=3, with 4 experimental replicates in each group).

(e) Effect of palbociclib on PI3K/AKT/mTOR activation. MCF-7-WT cells were treated with palbociclib (0.1 µM) or vehicle for 48 or 96 hours. Protein lysates were analyzed by immunoblot with the indicated antibodies. Palbociclib inhibited Rb phosphorylation, though it did not affect AKT or S6 phosphorylation.

Original blots/gels are presented in Supplementary Material, unprocessed western blots section.

Sample ID	Total number of sequenced reads	Total number of uniquely mapped non-duplicate reads	Total average coverage	Total number of covered bases	Median coverage (and range) per base	Percentage of targeted bases with coverage ≥10
T47D-WT	48764388	48700912	102X	7305136800	40 (21-49)	86
T47D-TR	53349932	53270293	112X	7990543950	43 (21-56)	87
T47D-PR	40676828	40622496	85X	6093374400	36 (20-47)	83
T47D-TPR	44303118	44239921	93X	6635988150	38 (21-49)	84

Supplementary Table S1. Whole exome sequencing was performed for the T47D variants. Table showing sequencing coverage and quality statistics for each sample.

SUPPLEMENTARY MATERIALS AND METHODS

Animal Study Design

In vivo animal experiments were performed at least twice using between three and six mice per experimental group in each replica. Each figure represents one of the independent replicas. The number of mice required for the experiments was estimated according to previous results and pilot tests that allow a 25% difference between the means of wild type and resistant groups or control and treatment groups, with a p value of 0.05. Animals were randomly and equally assigned to be inoculated with either wild type or resistant cells, as well as to control or treatment groups, and experiments were conducted in a controlled and non-blinded manner. Primary endpoints in animal studies

were prospectively selected. In the resistant cell model, the studies involving a long-term result include the initiation and the establishment of the tumor, according to previous results. The studies involving long-term treatments include the tumor regressive process, according to pilot tests.

In xenograft studies the endpoint was selected to have divergence between therapeutic responses, according to previous results and pilot tests, but with enough remnant tumor tissue to perform histological and protein examination.

The criteria used for excluding animals during the in vivo experiments was established a priori for animals in which the tumor developed with a growth rate lower or higher than 50% of the mean in the group.

Randomization was used to inoculate experimental animals with wild type and resistant cells. Once the tumors were established, tumor-bearing mice were allocated homogenously in each treatment group to avoid a bias in tumor size at time 0.

The strategy used to minimize potential confounders was to alternate the order of treatments and measurements between animal/cage and to distribute each tumor type and each treatment group in separate cages.

Animal operator and study supervisor were aware of the group allocation of animals at the different stages of the experiment (during the allocation, the conduct of the experiment, the outcome assessment, and the data analysis).

To reduce pain, suffering and distress, animals were manipulated by a skilled operator and revised periodically by a veterinary doctor of the Animal Facility.

Cell cycle analysis by flow cytometry

Cells were fixed, stained for DNA content analysis, acquired, and analyzed as indicated in the Materials and Methods section. The gating strategy was as follows: First, cells were gated in a FSC (forward side scatter) vs SSC (side scatter) dot plot, for an initial elimination of the cell doublets based on cell size and granularity. From this selection, a new dot plot using the height and width (FL-H vs FL-W) of the fluorescent parameter PerCP (610/10 BP filter, used for the detection of the propidium iodide emission) was

made. In this new dot plot, a second gate was made to eliminate the cell doublets based on the width of the fluorescent signal, usually those events with a DNA content near tetraploid, but with a higher width (FL-W) signal. From this new selection, the histogram for the propidium iodide fluorescence was obtained using a linear scale, and the fluorescence peak of the propidium iodide equivalent to a "diploid" DNA content set in the 50 value channel of the fluorescence axis, to be able to compare among histograms from different samples. Quantitative analysis of the cell cycle phases was performed with FlowJo software using the Watson Pragmatic algorithm.

Quantitative real-time PCR (qRT-PCR)

Total RNA was extracted using TRIzol reagent (Thermo Fisher Scientific), and cDNA was synthesized using RevertAid Reverse Transcriptase (Thermo Fisher Scientific) according to the manufacturer's protocols. qPCR was performed with FastStart Universal SYBR Green Master mix (Rox) (Roche, New Jersey, USA) using a Biorad C1000 Touch Thermal Cycler. The relative mRNA expression was normalized to GAPDH expression and determined using the ΔΔCt method. The primers used were NANOG: forward 5′-ATGCAACCTGAAGACGTGTG-3′ and reverse 5′-AGGCTCCAACCATACTCCAC-3′; OCT4: forward 5′-GGTCCGAGTGTGGTTCTGTA-3′ and reverse 5′-GGAAAGGGACCGAGGAGTAC-3′; BCRP, forward 5′-CAGGTCTGTTGGTCAATCTCACA-3′ and reverse 5′-TCCATATCGTGGAATGCTGAAG-3′; and GAPDH: forward 5′-GGAAGGTGAAGGTCGGAGTCA-3′ and reverse 5′-GTCATTGATGGCAACAATATCCACCT-3′.

PDXs generation

Tissue samples from breast cancer patients were implanted in immunosuppressed mice and the generated tumors were successively transplanted until the final establishment of PDXs. Next, PDX tumors were mechanically and enzymatically disaggregated to obtain single cells (PDCs) which were next grown on top of Matrigel. Cells were treated with inhibitors to analyze their sensitivity by spheroid area measurement or western blot analysis.

Experiments were conducted following the European Union's animal care directive (2010/63/EU) and were approved by the Ethical Committee of Animal Experimentation of the Vall d'Hebron Research Institute, the Catalan Government or by the National Research Ethics Service, Cambridgeshire and https://caldaslab.cruk.cam.ac.uk/bcape/.

PDCs isolation

PDX breast tumors were collected after surgical resection, minced using sterile scalpels, and dissociated for 60 min in DMEM/F12/HEPES (GIBCO) supplemented with 1 mg/ml collagenase (Roche), 100 u/ml hyaluronidase (Sigma-Aldrich), 5% bovine serum albumin (Sigma-Aldrich), 5 µg/ml insulin, and 50 µg/ml gentamycin (GIBCO). Further dissociation was performed using trypsin (GIBCO), dispase 5 mg/ml (StemCell technologies, Vancouver, British Columbia, Canada) and DNase 1 mg/ml (Sigma-Aldrich). Cell pellets were treated with red blood cell (RBC) lysis buffer (Invitrogen) and resuspended in DMEM/F12 with 2% charcoal-stripped FBS containing 10 µg/ml ROCK inhibitor, 3 ng/ml EGF, 5 µg/ml insulin, 1 µg/ml hydrocortisone, B-27 supplement 1X (GIBCO), 30 µg/ml gentamicin, and 15 ng/ml fungizone.

Immunohistochemistry

Tumor and tissue histology were evaluated by hematoxylin and eosin staining. For immunostaining, formalin-fixed paraffin-embedded tissue sections were deparaffinized and rehydrated, endogenous peroxidase was blocked, and antigen retrieval was performed using a sodium citrate buffer (pH 6.0). The sections were then blocked with 2,5% bovine serum albumin for 1 hour at room temperature, followed by incubation with primary antibodies at 4°C overnight. Slides were then incubated with biotinylated secondary antibodies (Vector Laboratories) for 1 hour at room temperature and for 30 min with the Vectastain Elite ABC Kit (Vector Laboratories). Staining was performed using diaminobenzidine tetrahydrochloride solution (Vector Laboratories), followed by counterstaining with hematoxylin (Biopur, Santa Fe, Argentina), air-drying, and mounting with DPX (Sigma). Images were obtained with a Nikon Eclipse E800 microscope using the ACT-2U software.

Sequencing experiments

DNA extraction and library construction for Whole Exome Sequencing (WES)

Genomic DNA was isolated using the EasyPure Genomic DNA Kit (TransGen Biotech, Beijing, China), following the manufacturer's instructions. Quality control of DNA samples included agarose gel electrophoresis and DNA quantification using the QuantiFluor dsDNA System and Quantus equipment (Promega, Madison, Wisconsin, USA). 1.0 μg of genomic DNA per sample was used as input material for library preparation. For WES, libraries were generated using the Agilent SureSelect Human All ExonV6 kit (Agilent Technologies, Santa Clara, California, USA), following the manufacturer's recommendations. The synthesized sequencing libraries were purified using the AMPure XP System (Beckman Coulter, Brea, California, USA) and quantified using the Agilent High Sensitivity DNA Kit (Agilent Technologies) and the Agilent Bioanalyzer 2100 System (Agilent Technologies). NGS sequencing runs were made in Illumina Novaseq systems as an outsourced service and 150-bp paired-end reads were generated.

Sequencing data analysis

Quality control of the raw sequencing data was performed. Specifically, the sequencing quality, sequencing error rate, and A/T/G/C base distribution were evaluated. Data filtering was performed as follows: 1) removing reads that contained adapters, 2) removing reads that contained over 10% of undetermined bases (N > 10%), and 3) eliminating reads that contained 50% of bases with low quality (Q score less than 5).

Pre-processed sequence data in FastQ format were aligned to the reference genome GRCh38 (NCBI, NIH, USA) using the Burrows-Wheeler alignment tool (BWA-MEM algorithm). After realignment, the Picard software was used to mark duplicate reads. An overview of the sequencing data is provided in **Supplementary Table 1.**

The "Variant Calling and Filtering" step was performed using version 4.1.4.0 of the Genomic Analysis Toolkit (GATK) following best practices. To detect single nucleotide

variants (SNV) and small insertions/deletions (INDELs) of the germinal type, HaplotypeCaller was used. Somatic variants were called using MuTect.

Subsequently, the output VCF file was annotated at various levels using ANNOVAR with information from several databases. The variants were prioritized based on pathogenicity using the predictors REVEL, SIFT, Polyphen2, and CLNSIG.

Variants were classified according to the recommendations of the American College of Medical Genetics and Genomics (ACMG).

Antibodies

ERα (sc-543), PGR (sc-7208), cyclin A (sc-751), CDK2 (sc-163), PI3K (sc-376641), pERK T204 (sc-7383), ERK 1/2 (sc-514302), E-cadherin (sc-7870), actin (sc-47778) were purchased from Santa Cruz Biotechnology, Dallas, Texas, USA; pRb S807/811 (#8516), Rb (#9309), cyclin E1 (#4129), cyclin E2 (#4132), cyclin B1 (#4138), CDK4 (#12790), CDK6 (#3136), pPTEN S380/T382/383 (#9554), PTEN (#9188), pAKT T308 (#4056), pAKT S473 (#4060), AKT (#4685), pS6 S240/244 (#2215), pS6 S235/236 (#2211), S6 (#2217), vinculin (#13901) from Cell Signaling Technology, Danvers, Massachusetts, USA; actin (A2066), β-tubulin (T0198 clone D66) from Sigma; cyclin D1 (#RM-9104-S1), CDK1 (#33-1800) from Thermo Fisher Scientific; and PKCα (#610107) from BD Transduction Laboratories, San Diego, California, USA.
