# Supplementary material for: Targeting mTOR to overcome resistance to hormone and CDK4/6 inhibitors in ER-positive breast cancer models
Source: Sci Rep. 2023 Feb 15;13:2710. doi: 10.1038/s41598-023-29425-y (PMC9932145; doi:10.1038/s41598-023-29425-y)

Uncropped, unprocessed images

Figure 2

a

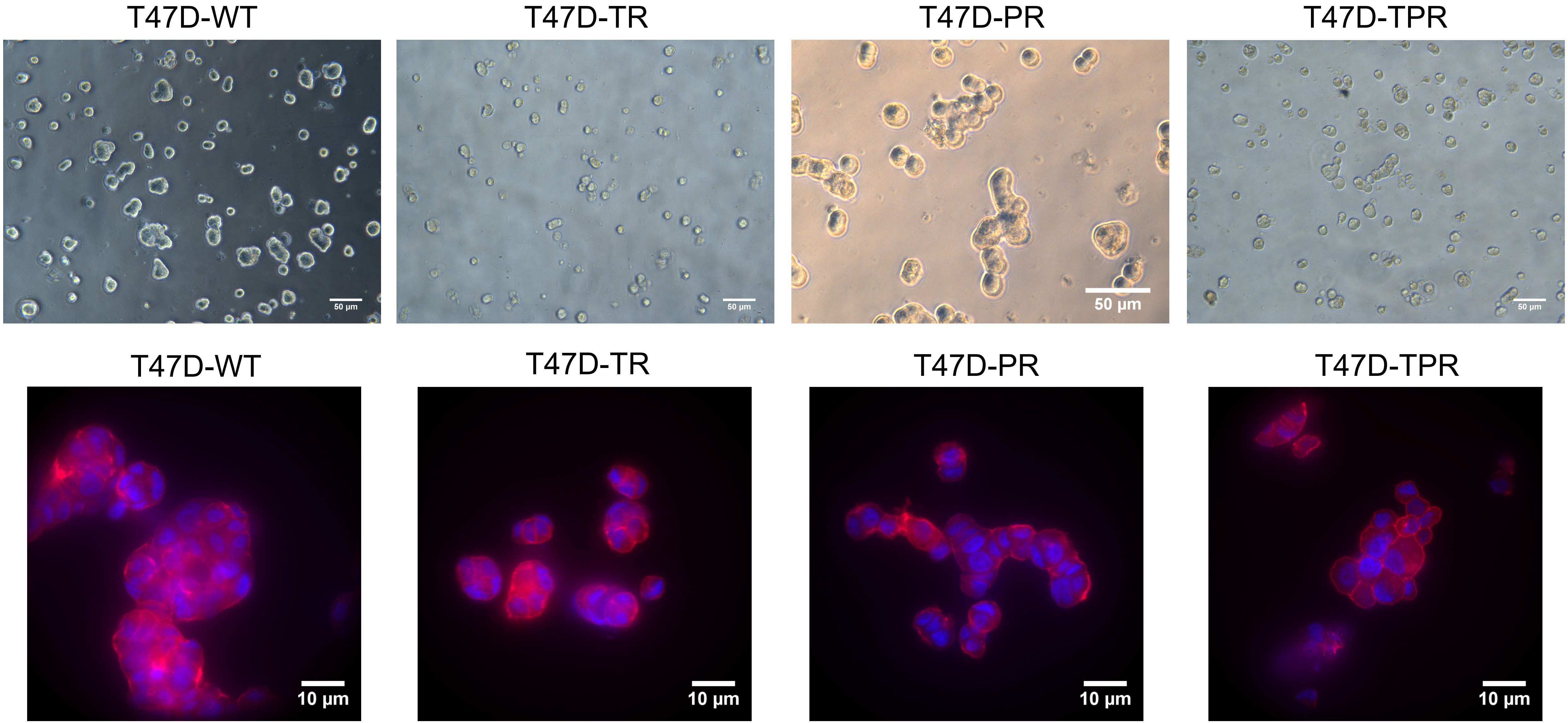

Figure 2 (merged)

b

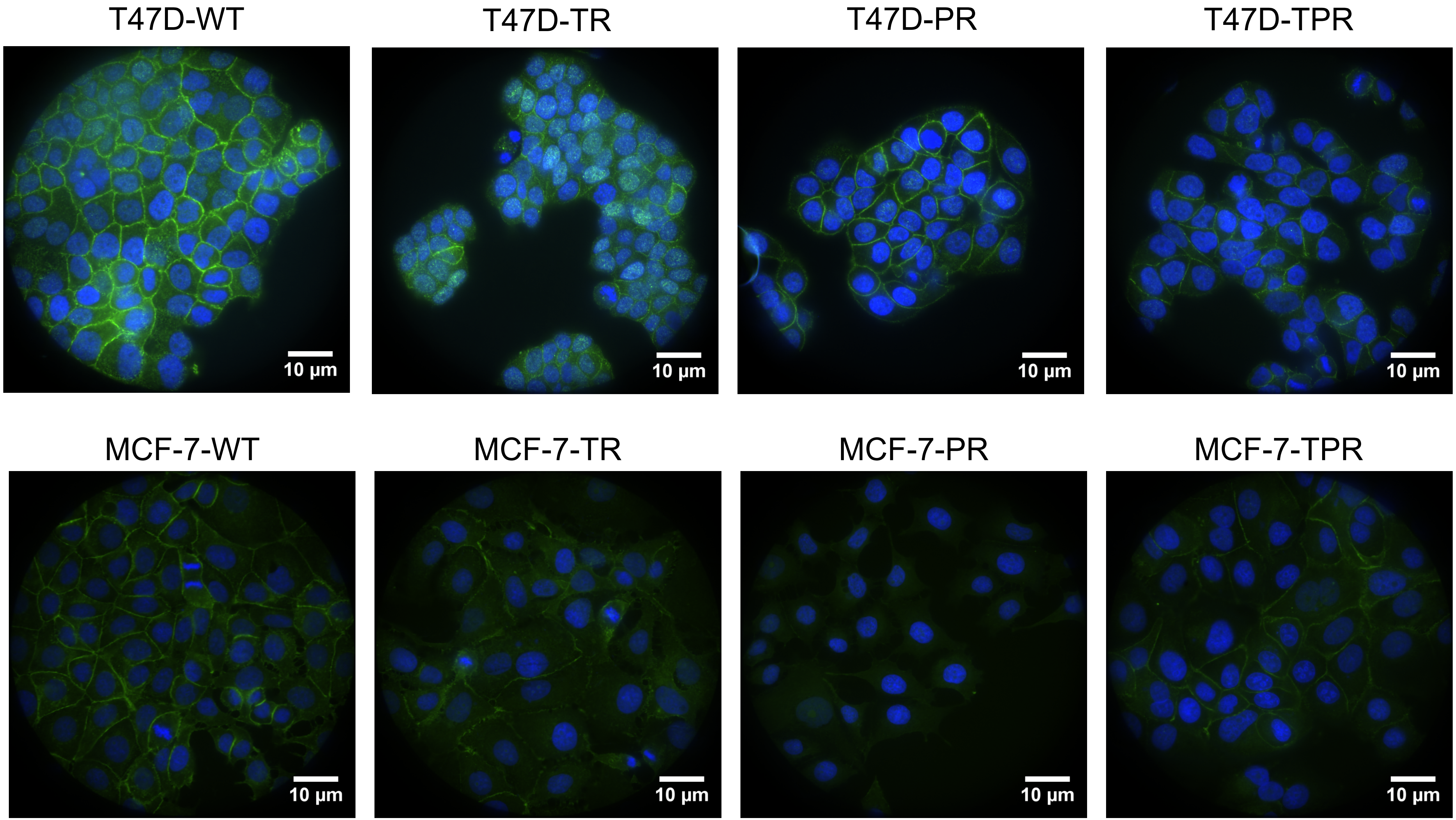

Figure 2 (E-cadherin)

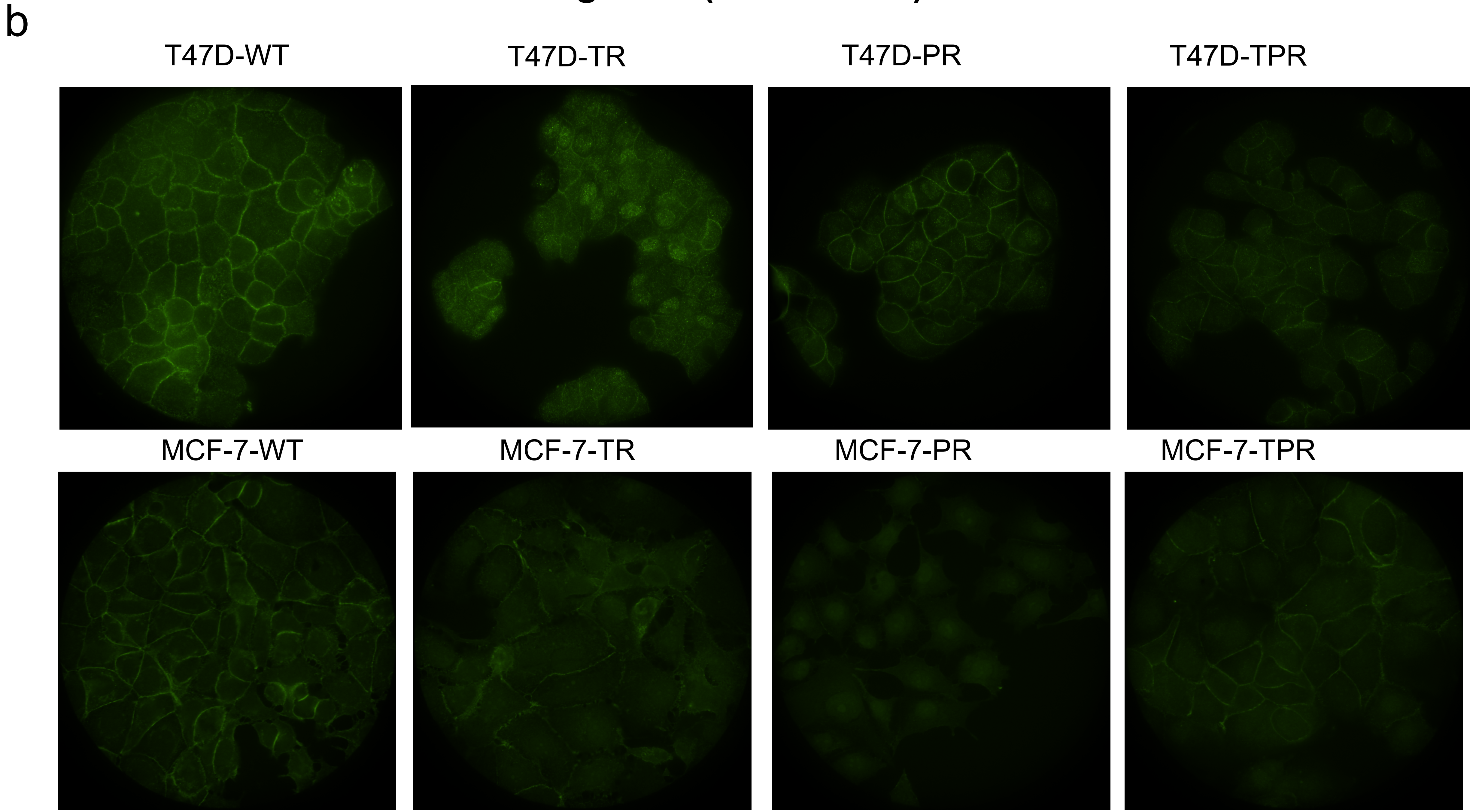

Figure 2 (dapi)

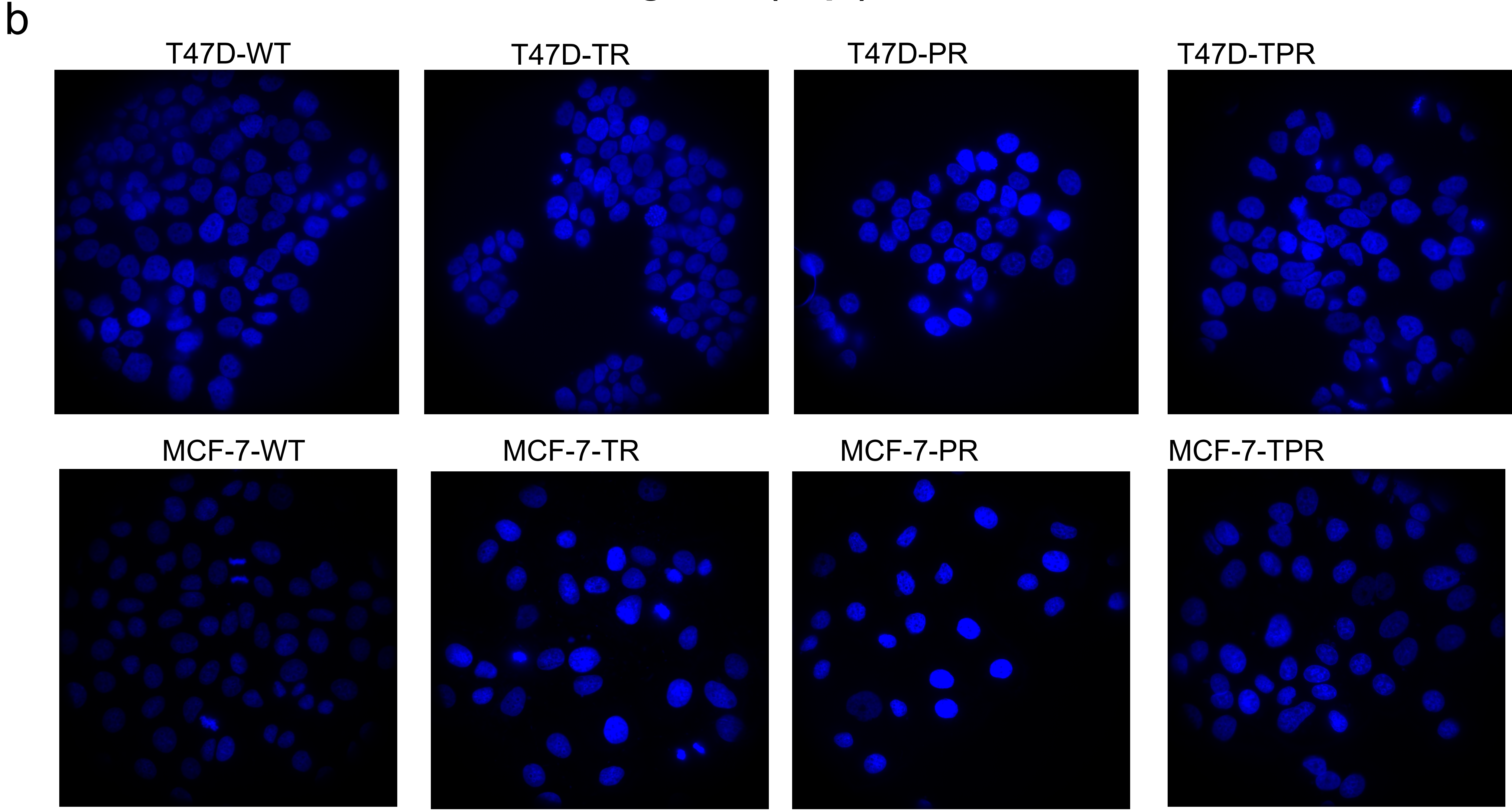

Figure 2

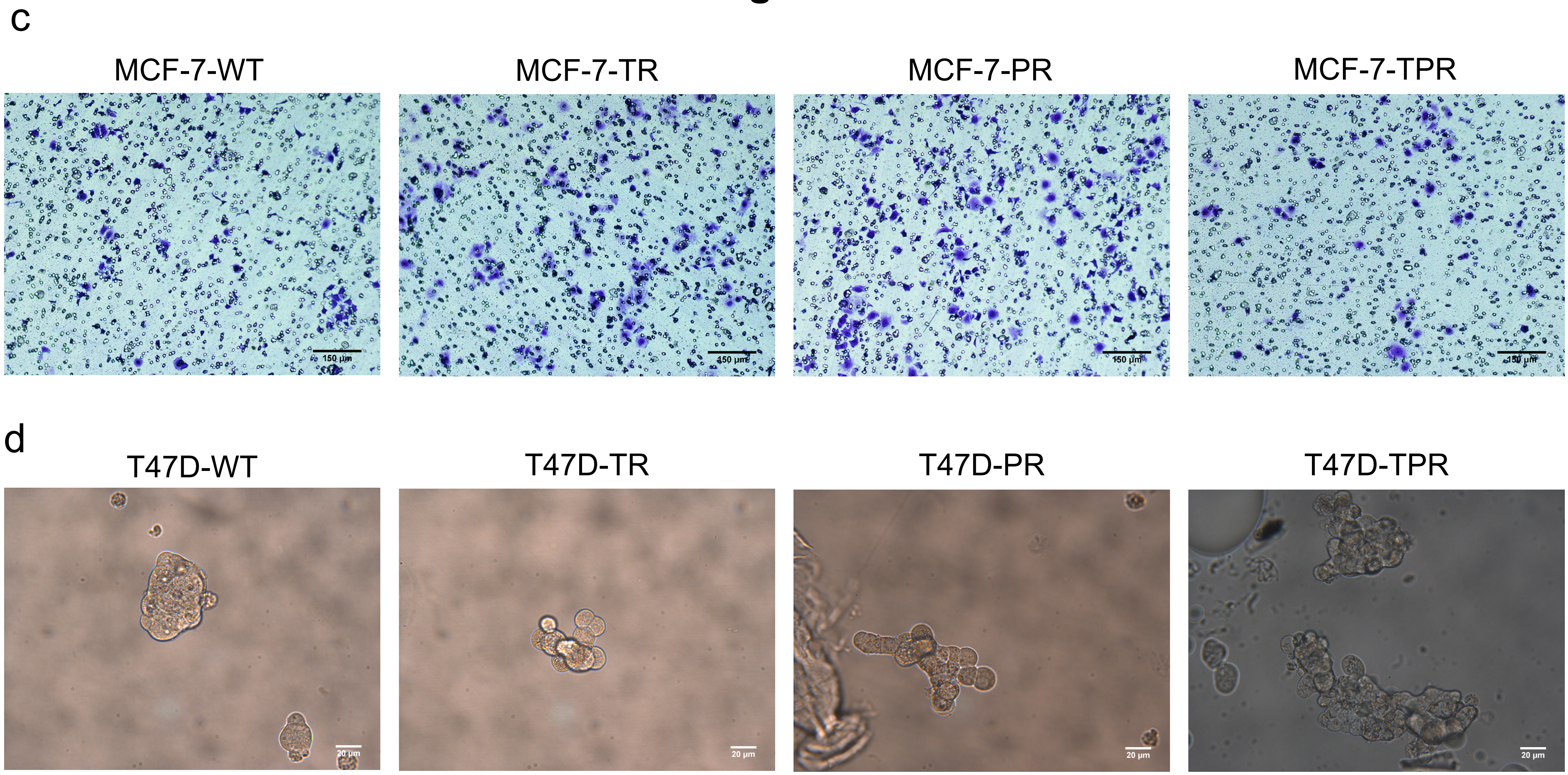
c
MCF-7-WT
MCF-7-TR
MCF-7-PR
MCF-7-TPR
150 μm
150 μm
150 μm
150 μm
d
T47D-WT
T47D-TR
T47D-PR
T47D-TPR
20 μm
20 μm
20 μm
20 μm

Figure 3

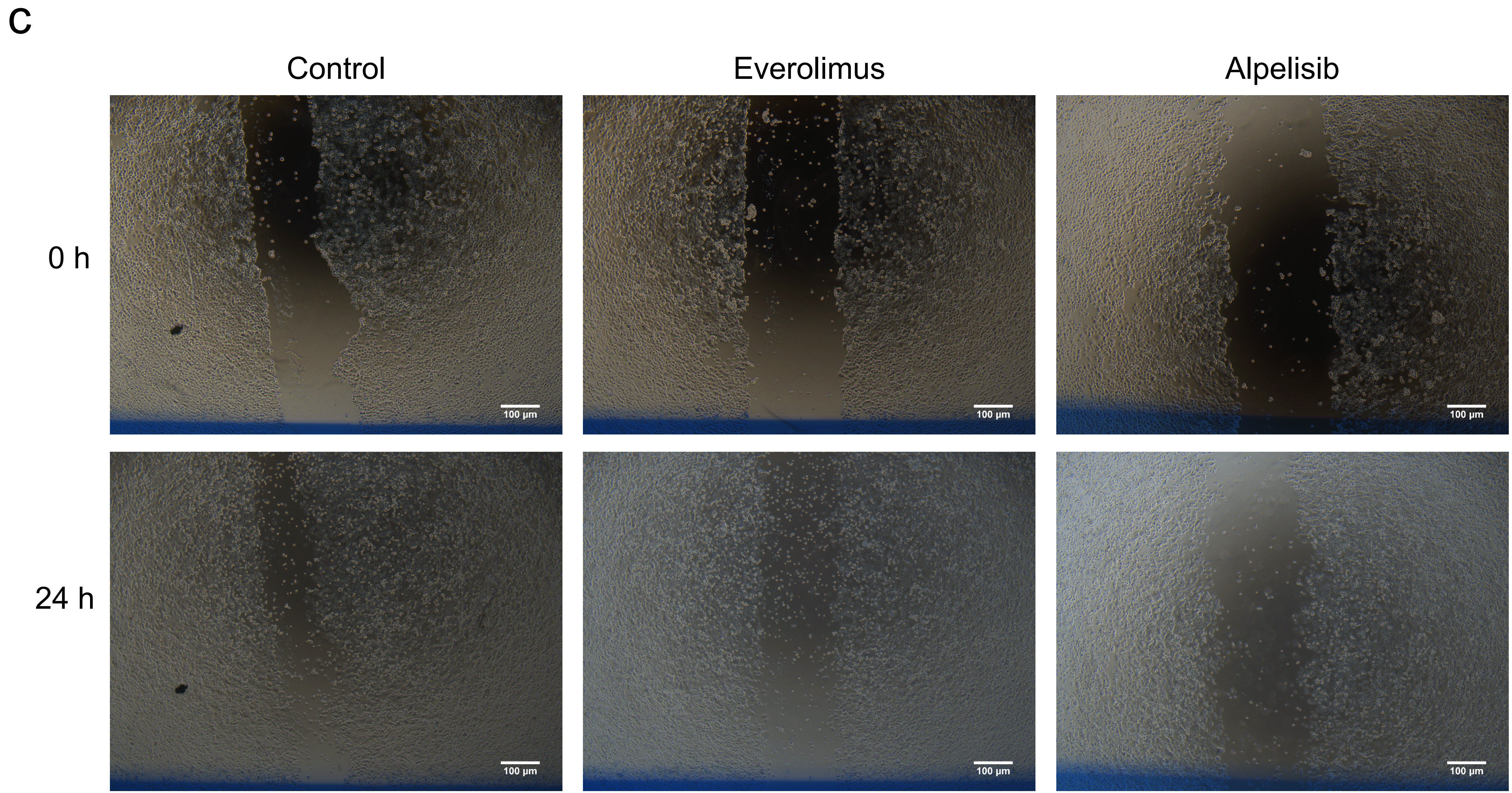
C
Control
Everolimus
Alpelisib
0 h
24 h
100 µm
100 µm
100 µm
100 µm
100 µm
100 µm

Figure 4

d

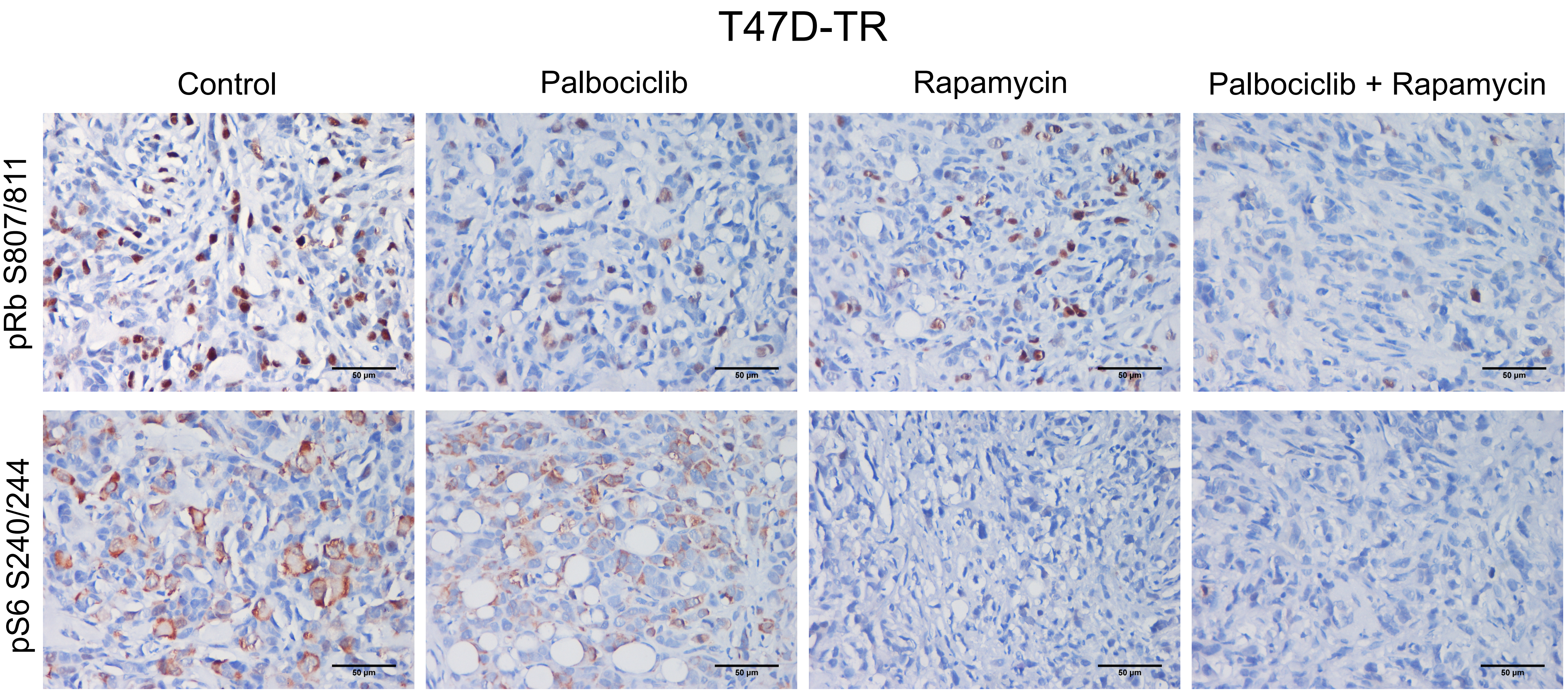
T47D-TR
Control
Palbociclib
Rapamycin
Palbociclib + Rapamycin
pRb S807/811
pS6 S240/244
50 μm

Figure 4

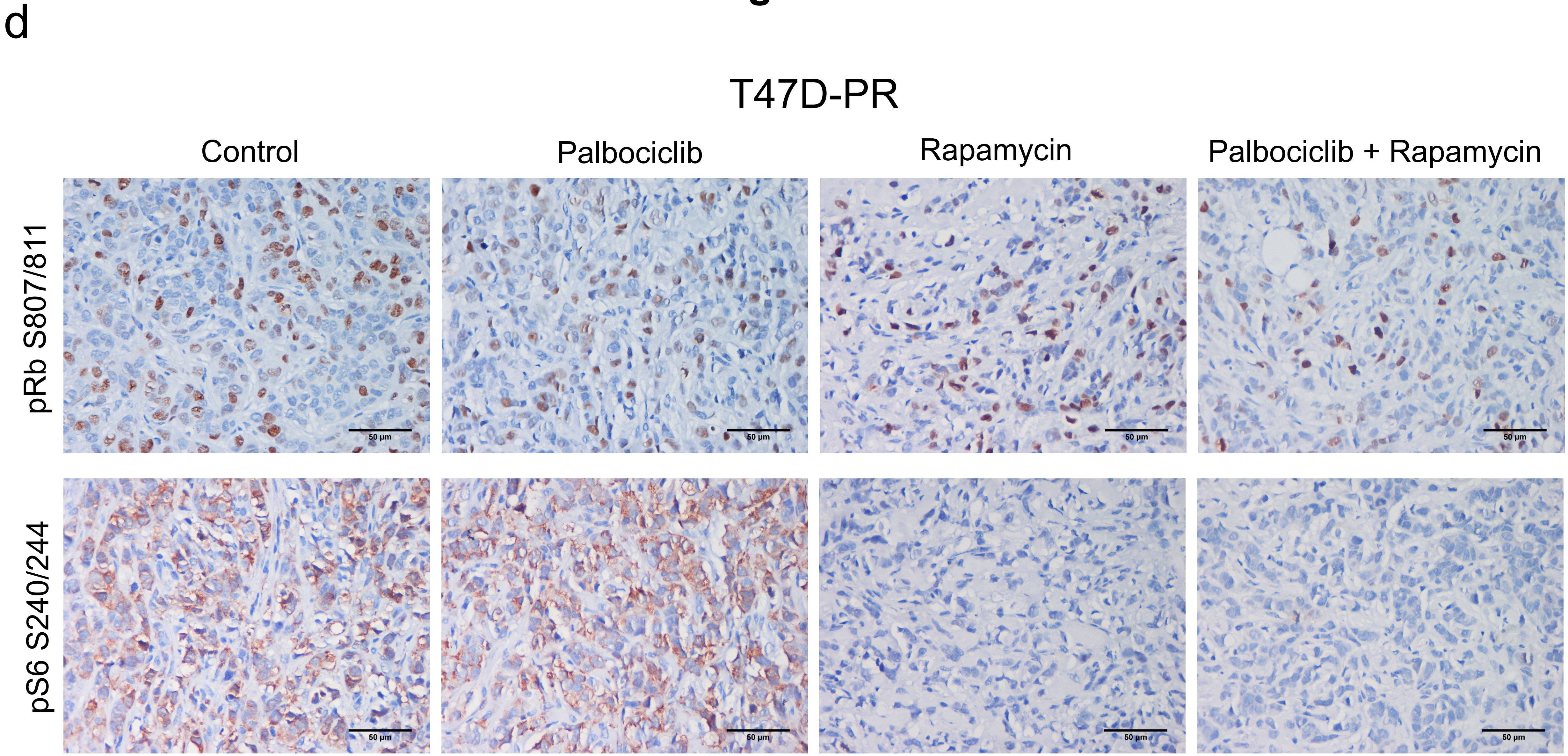
d
T47D-PR
Control
Palbociclib
Rapamycin
Palbociclib + Rapamycin
pRb S807/811
pS6 S240/244
50 μm

Supplementary Figure 1

d

T47D-PR

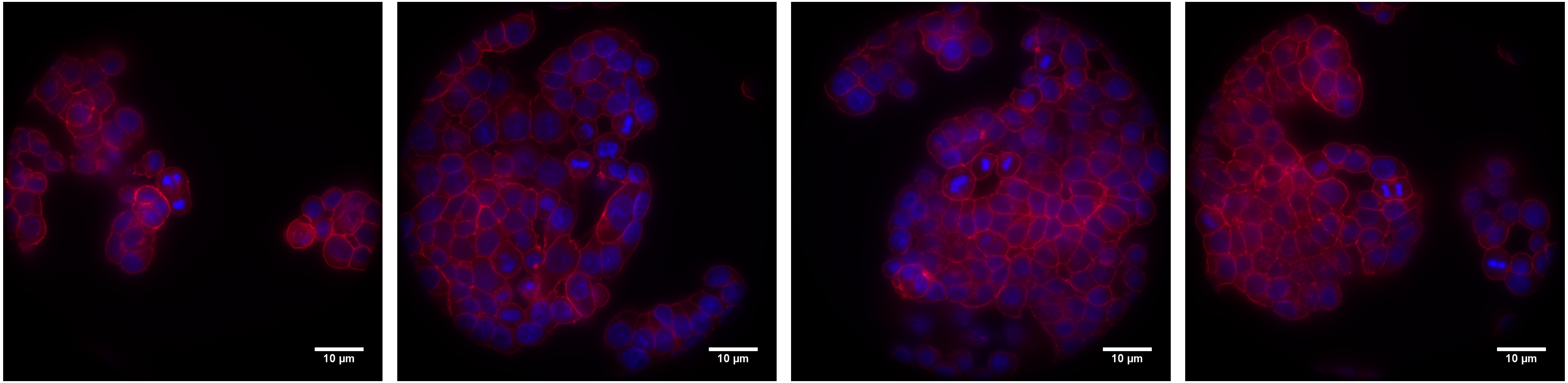

Supplementary Figure 1

e

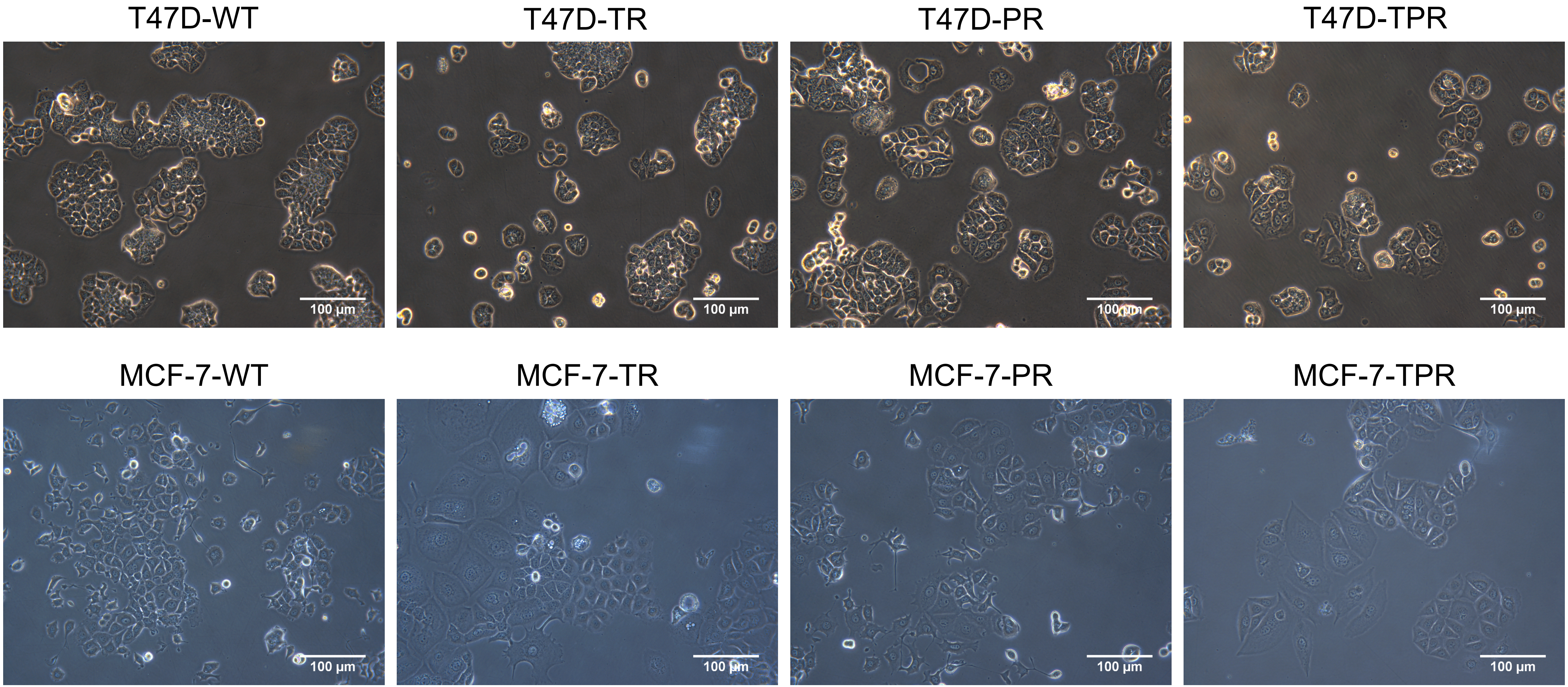

Supplement: Supplementary file 2 — Supplementary Information 2. [file 41598_2023_29425_MOESM2_ESM.pdf]
