# Supplementary material for: Targeting mTOR to overcome resistance to hormone and CDK4/6 inhibitors in ER-positive breast cancer models
Source: Sci Rep. 2023 Feb 15;13:2710. doi: 10.1038/s41598-023-29425-y (PMC9932145; doi:10.1038/s41598-023-29425-y)

Figure 1b

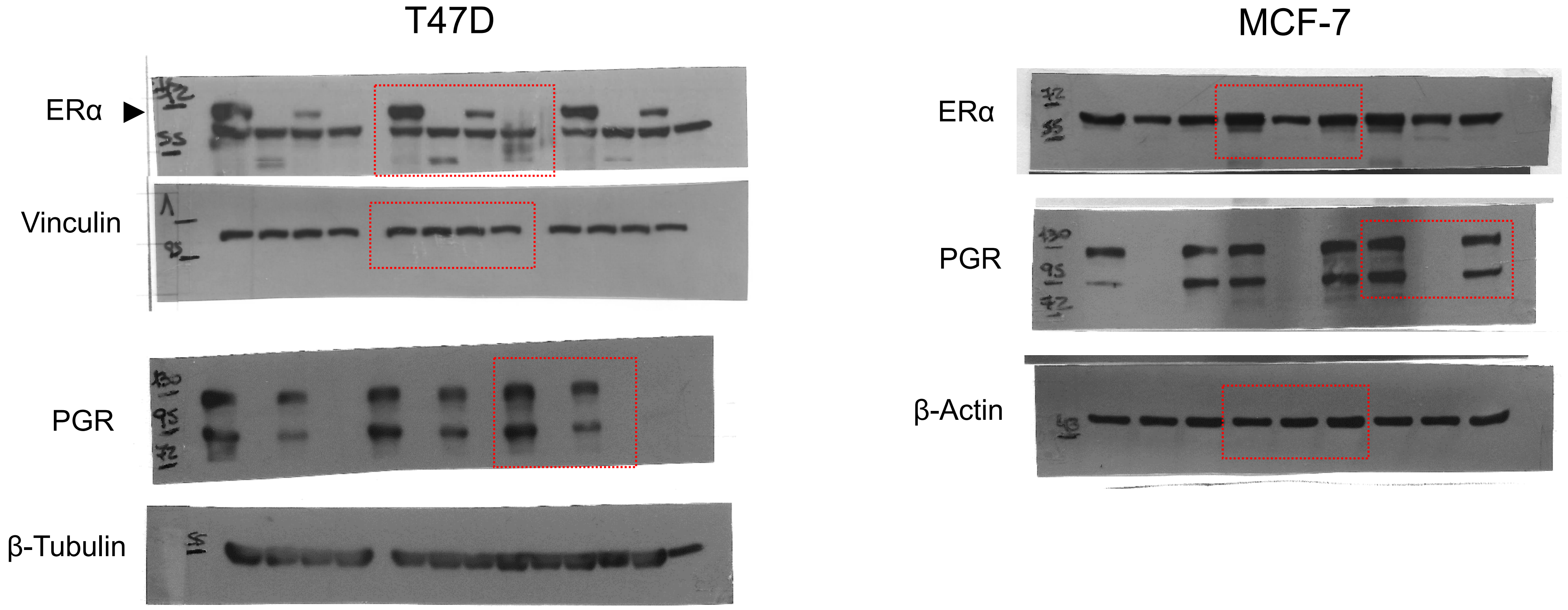

Figure 1f

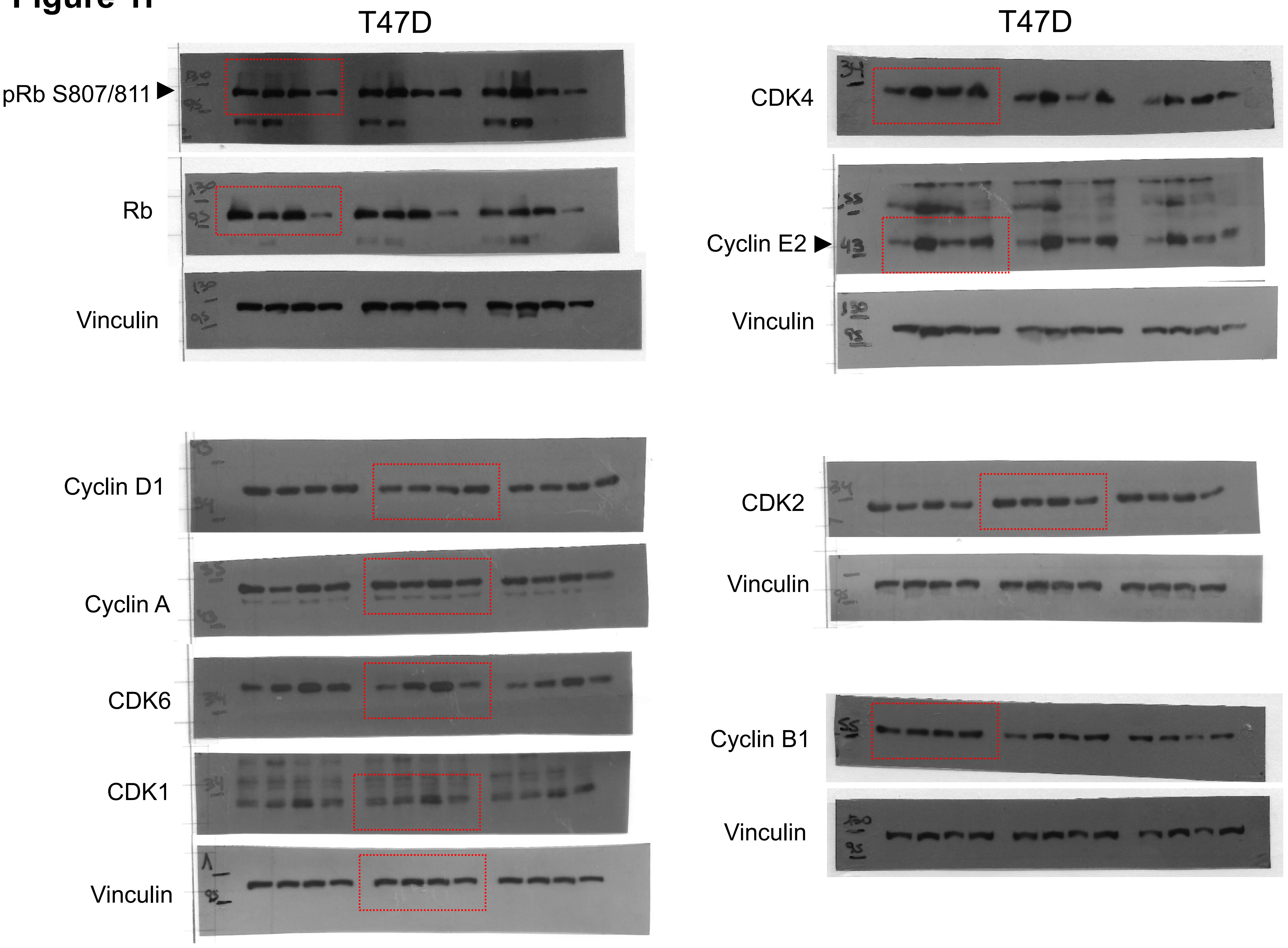

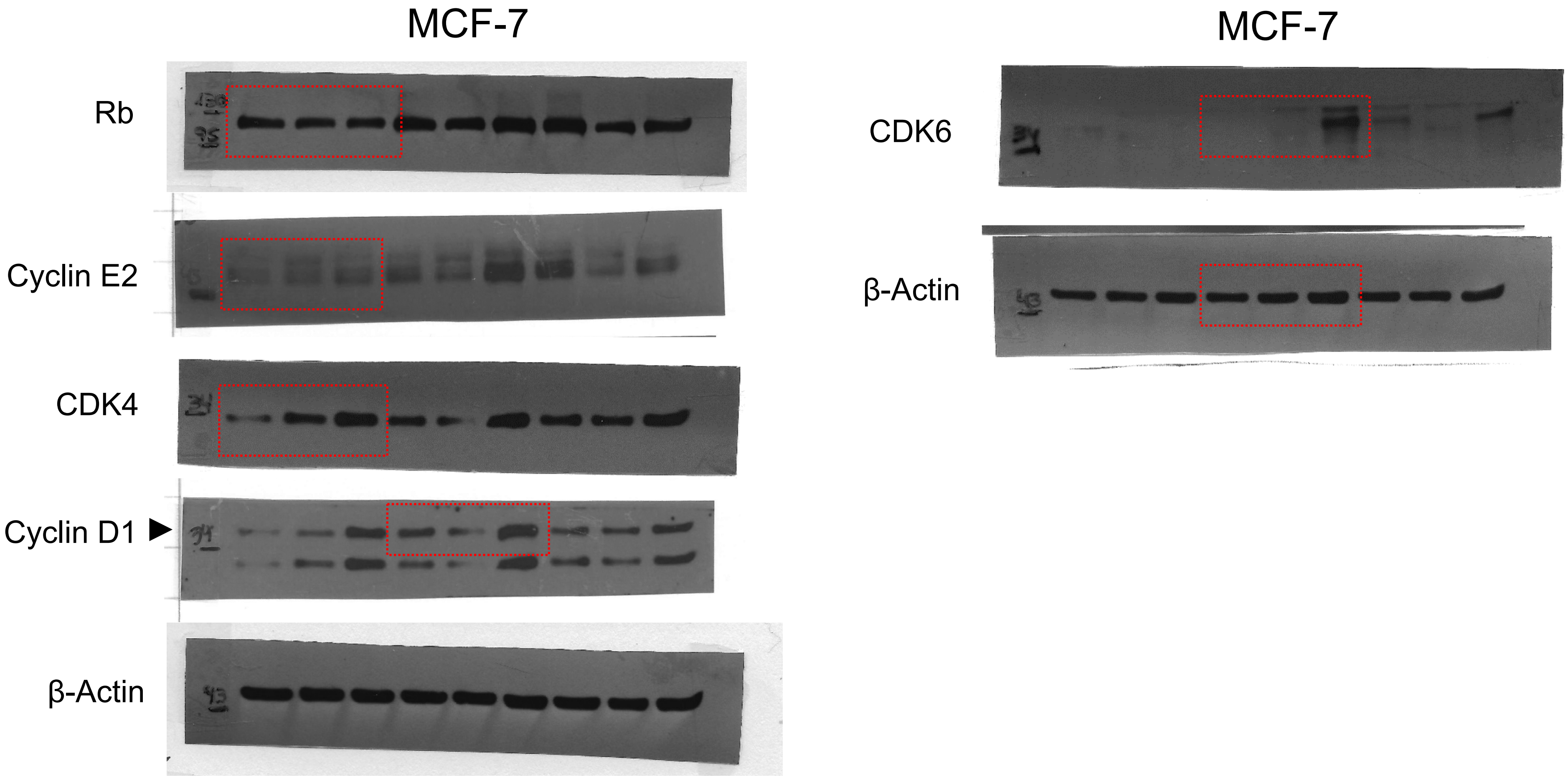
MCF-7
Rb
Cyclin E2
CDK4
Cyclin D1 ▶
β-Actin
MCF-7
CDK6
β-Actin

Figure 3a

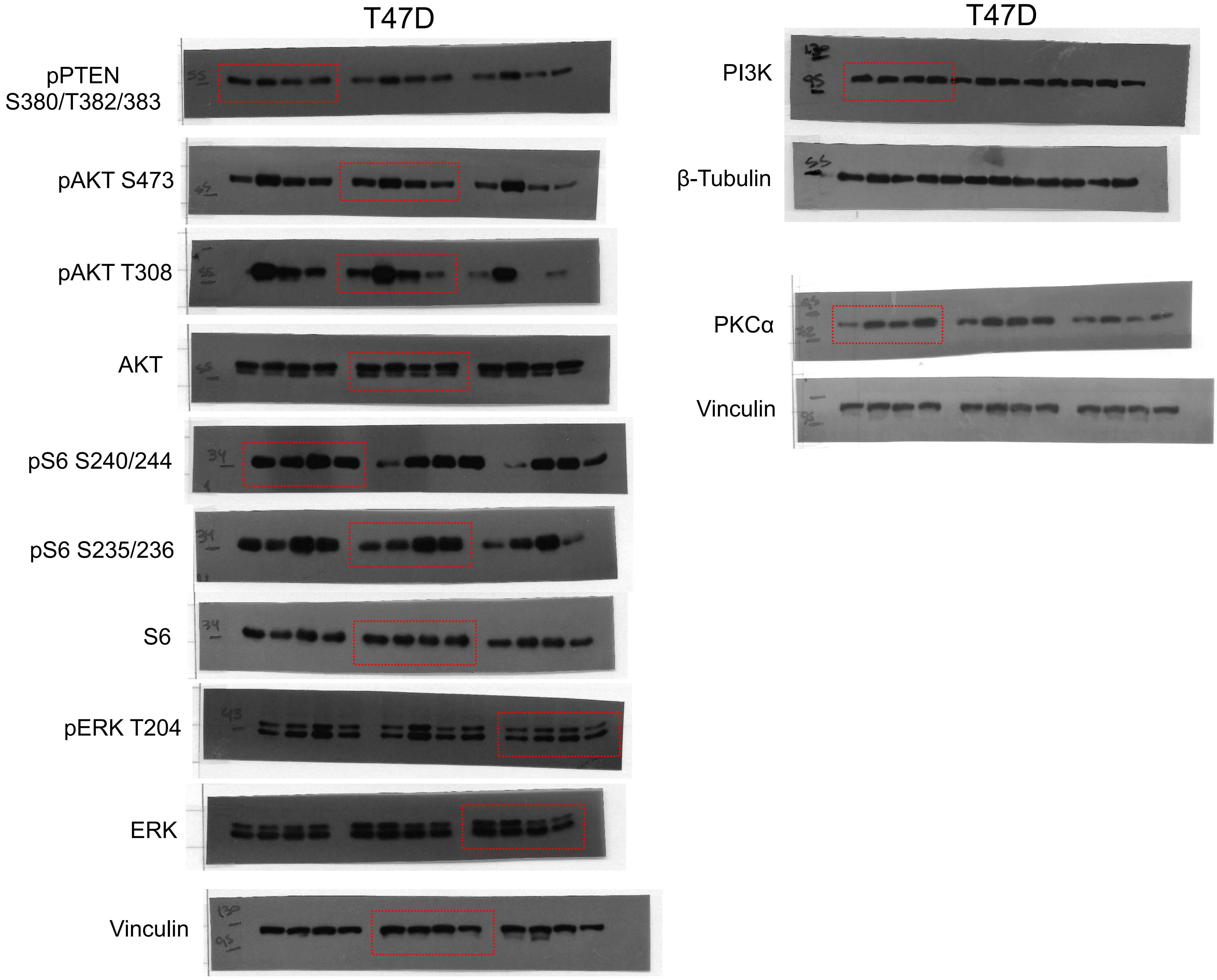
T47D
pPTEN S380/T382/383
pAKT S473
pAKT T308
AKT
pS6 S240/244
pS6 S235/236
S6
pERK T204
ERK
Vinculin
T47D
PI3K
β-Tubulin
PKCα
Vinculin

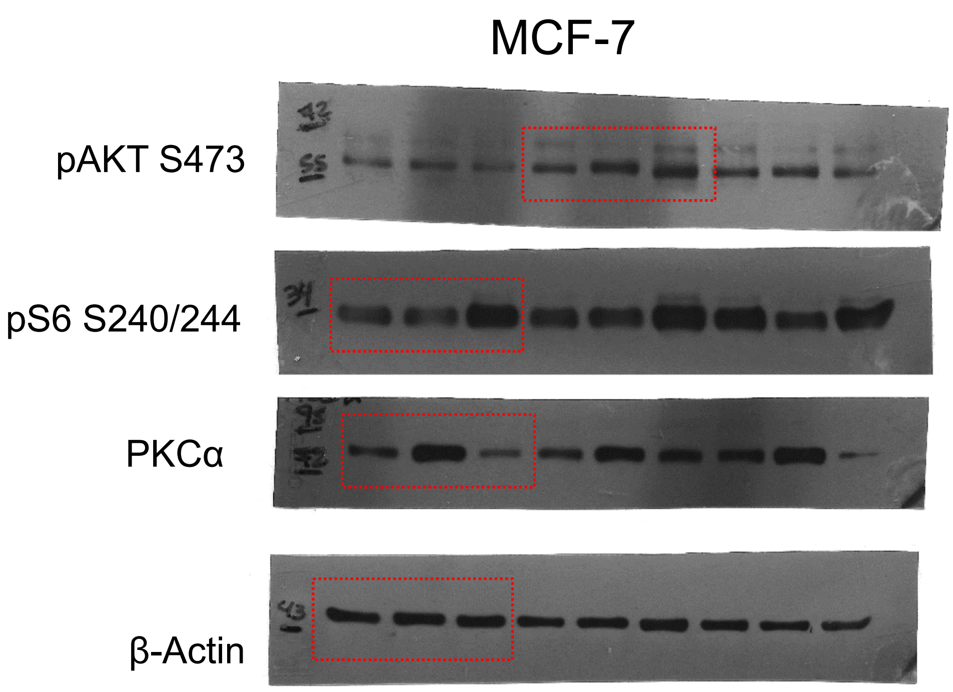
MCF-7
pAKT S473
pS6 S240/244
PKCα
β-Actin

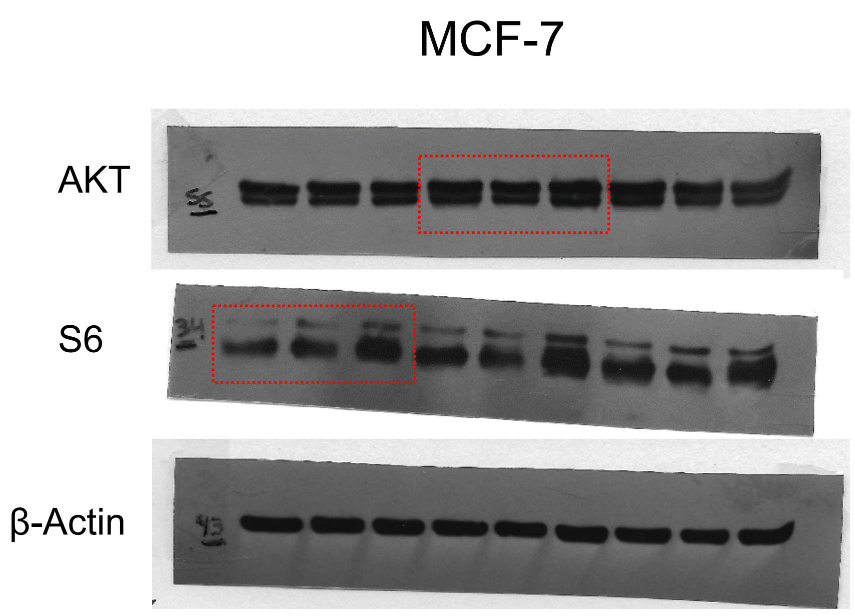
MCF-7
AKT
S6
β-Actin

Figure 4a

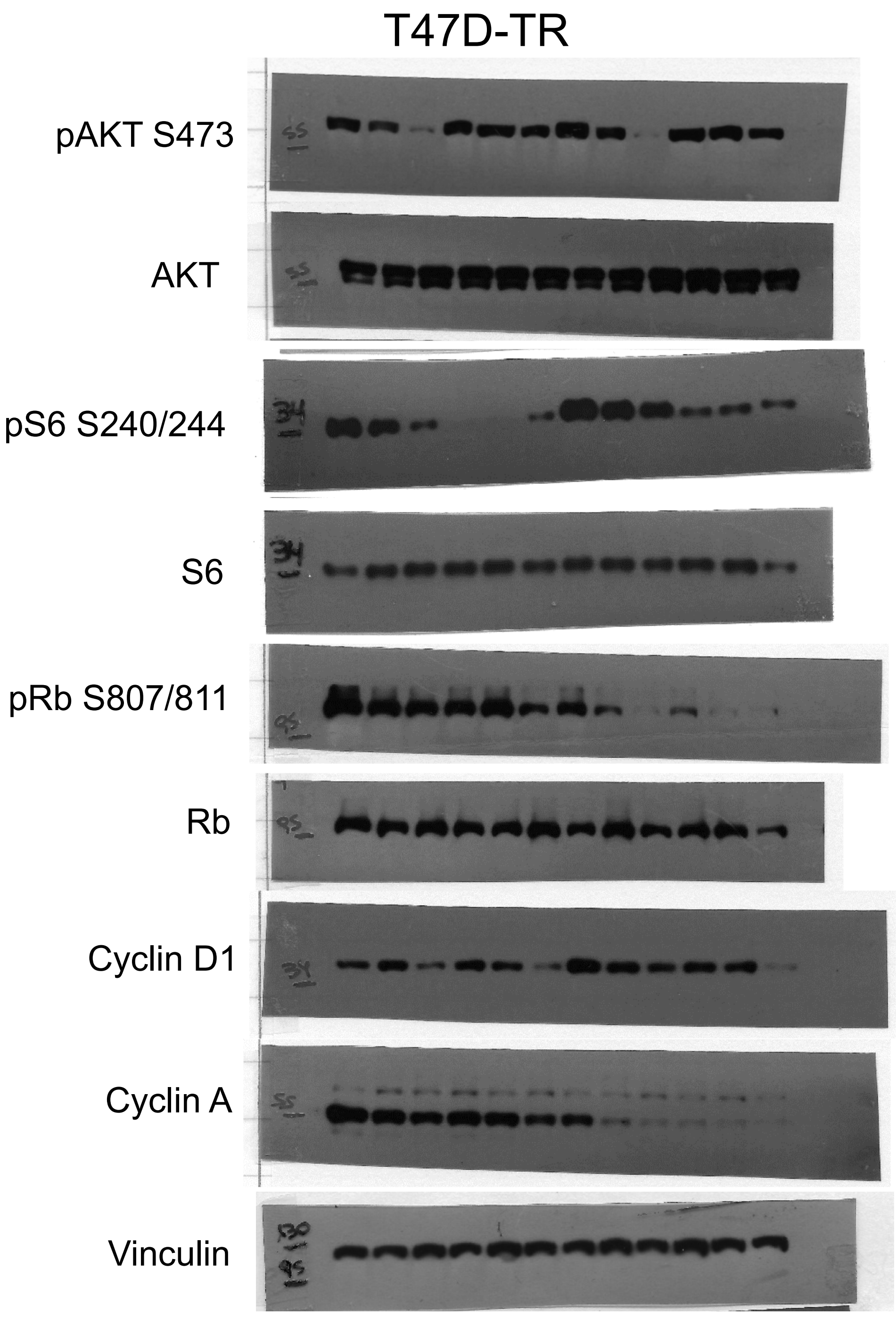
T47D-TR
pAKT S473
AKT
pS6 S240/244
S6
pRb S807/811
Rb
Cyclin D1
Cyclin A
Vinculin

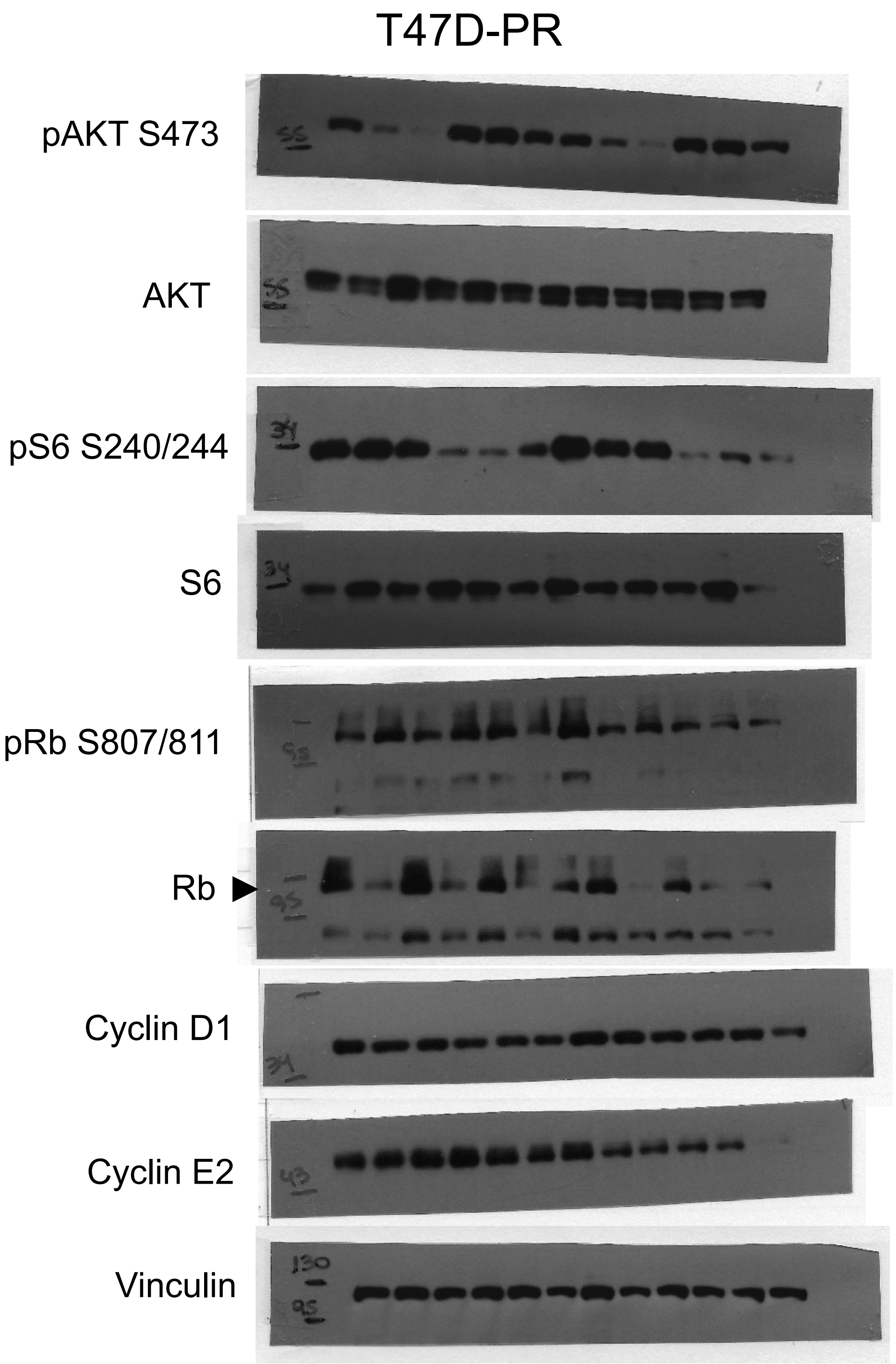
T47D-PR
pAKT S473
AKT
pS6 S240/244
S6
pRb S807/811
Rb
Cyclin D1
Cyclin E2
Vinculin

Figure 4e

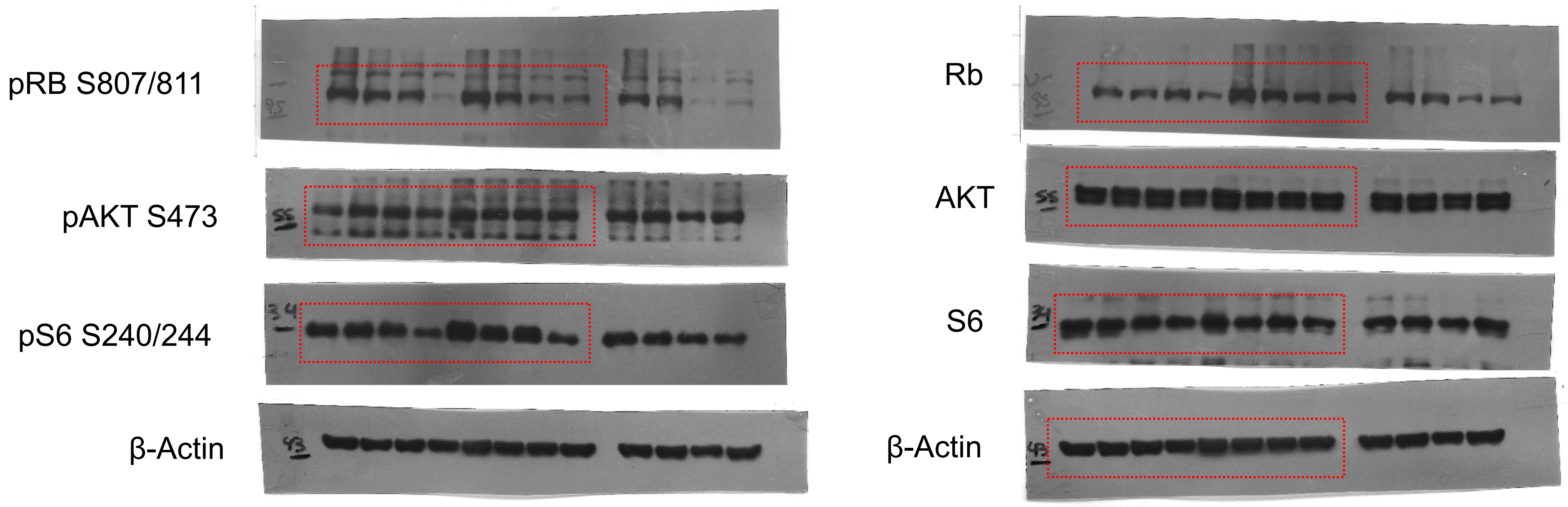

Figure 5d

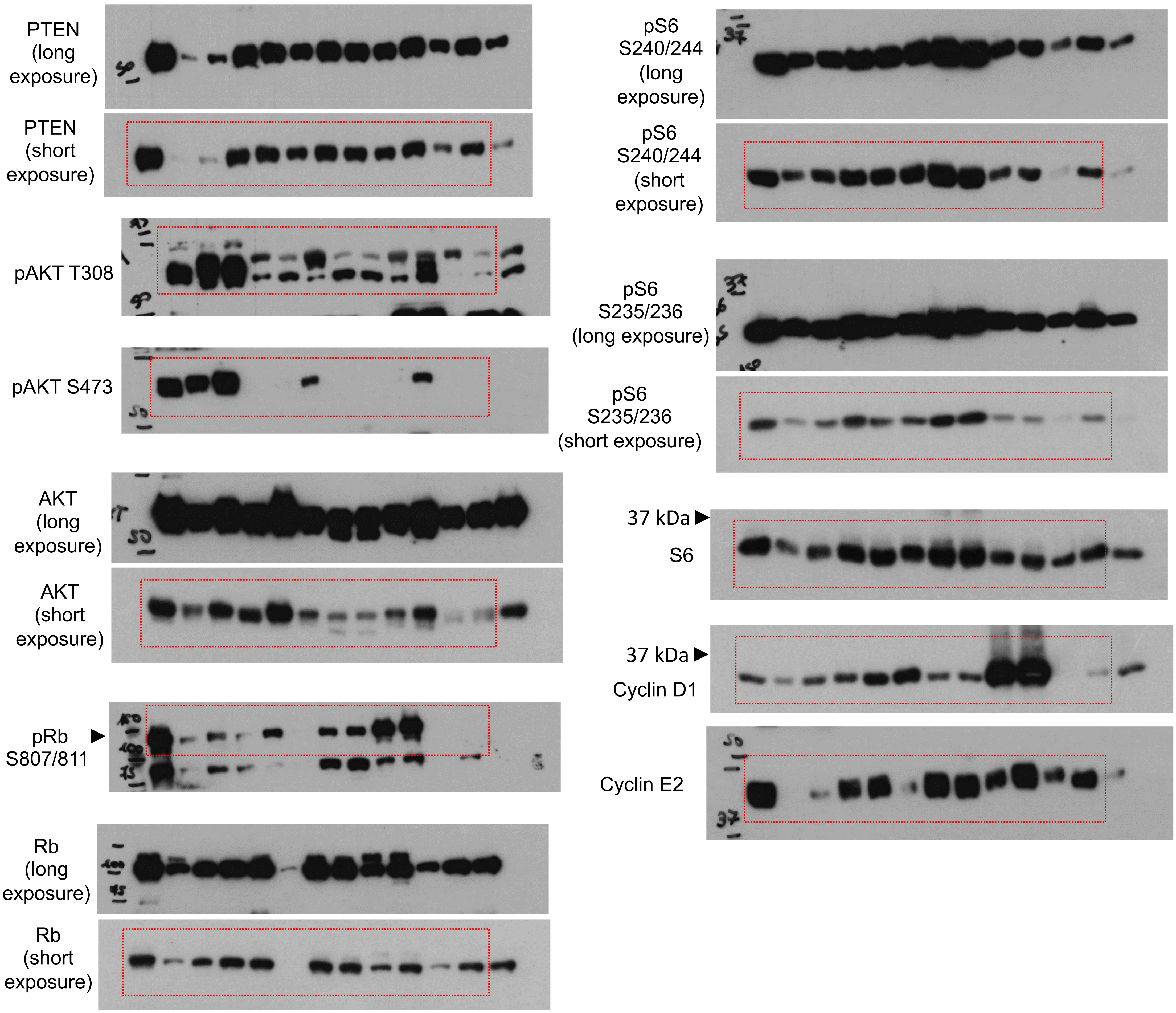

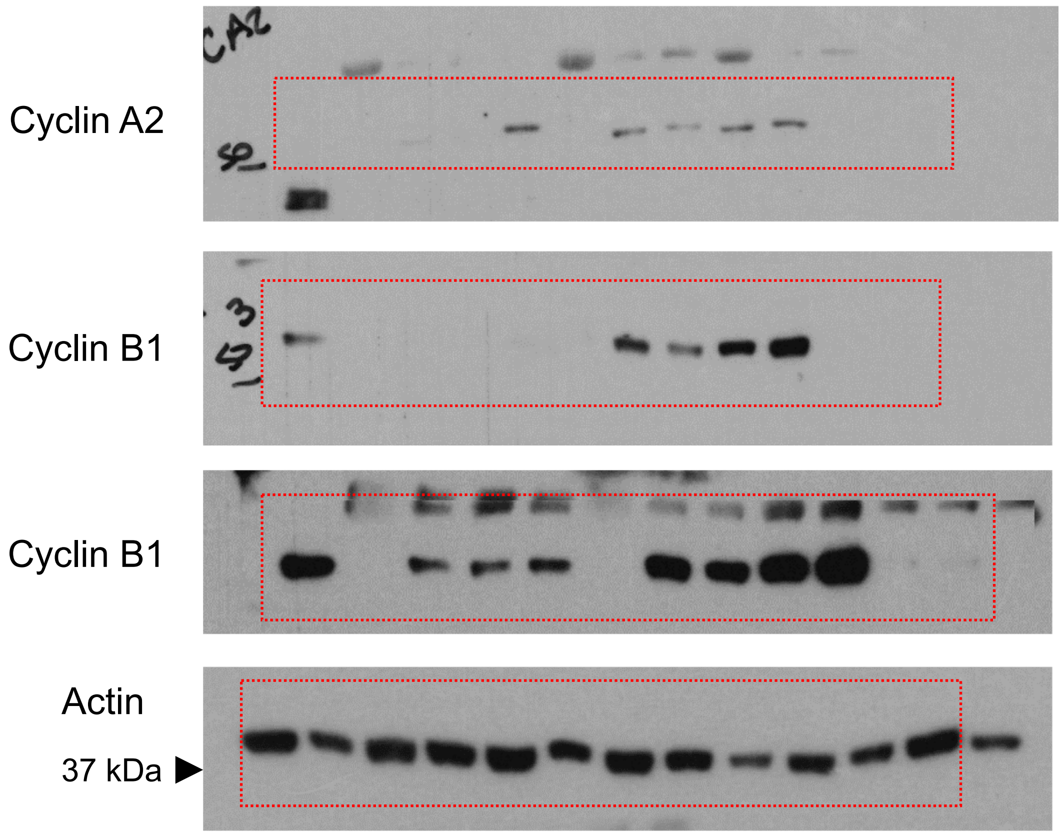
Cyclin A2
Cyclin B1
Cyclin B1
Actin
37 kDa

Figure 5g

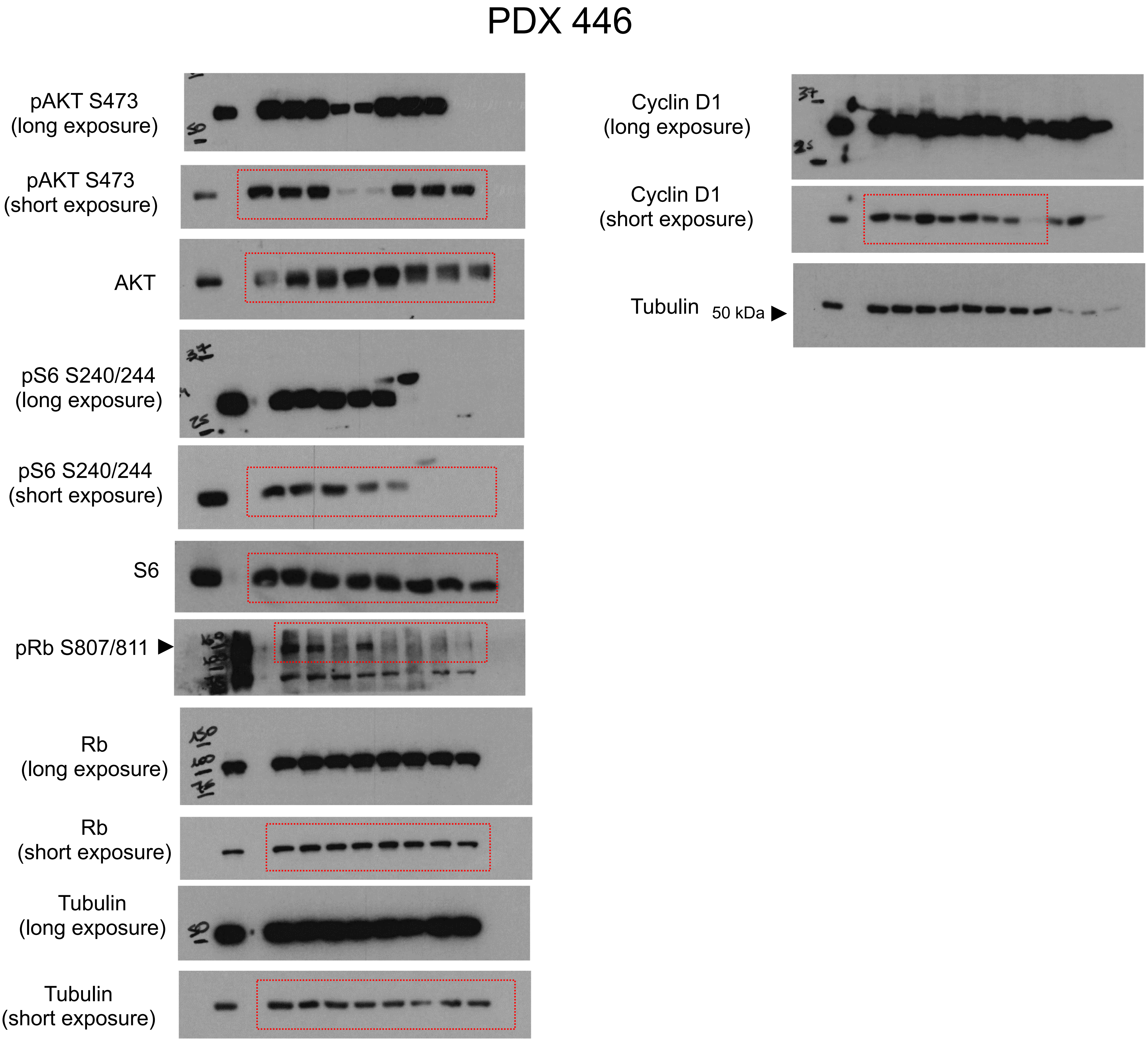
PDX 446
pAKT S473
(long exposure)
pAKT S473
(short exposure)
AKT
pS6 S240/244
(long exposure)
pS6 S240/244
(short exposure)
S6
pRb S807/811
Rb
(long exposure)
Rb
(short exposure)
Tubulin
(long exposure)
Tubulin
(short exposure)
Cyclin D1
(long exposure)
Cyclin D1
(short exposure)
Tubulin
50 kDa

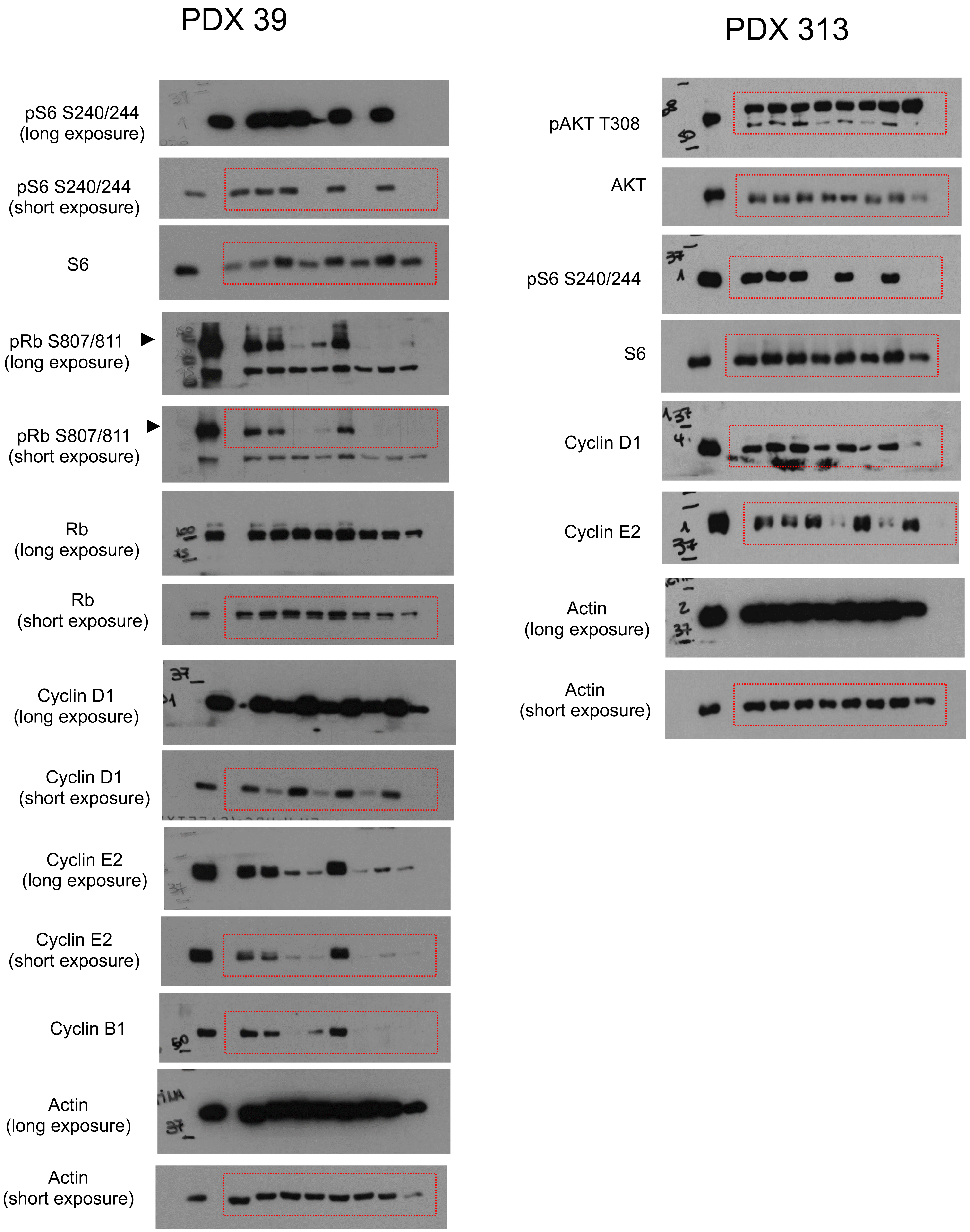
PDX 39
pS6 S240/244
(long exposure)
pS6 S240/244
(short exposure)
S6
pRb S807/811
(long exposure)
pRb S807/811
(short exposure)
Rb
(long exposure)
Rb
(short exposure)
Cyclin D1
(long exposure)
Cyclin D1
(short exposure)
Cyclin E2
(long exposure)
Cyclin E2
(short exposure)
Cyclin B1
Actin
(long exposure)
Actin
(short exposure)
PDX 313
pAKT T308
AKT
pS6 S240/244
S6
Cyclin D1
Cyclin E2
Actin
(long exposure)
Actin
(short exposure)

Supplementary Figure 2a

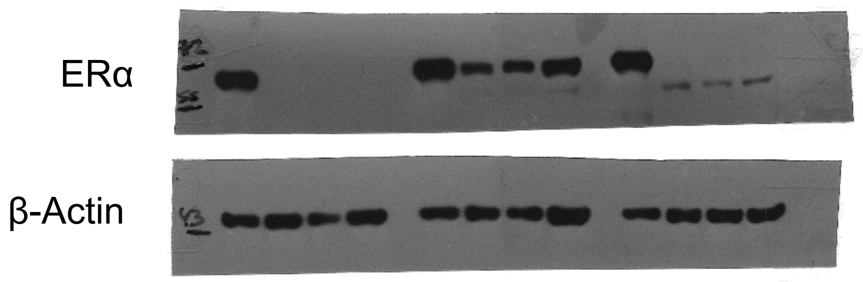

Supplementary Figure 2b

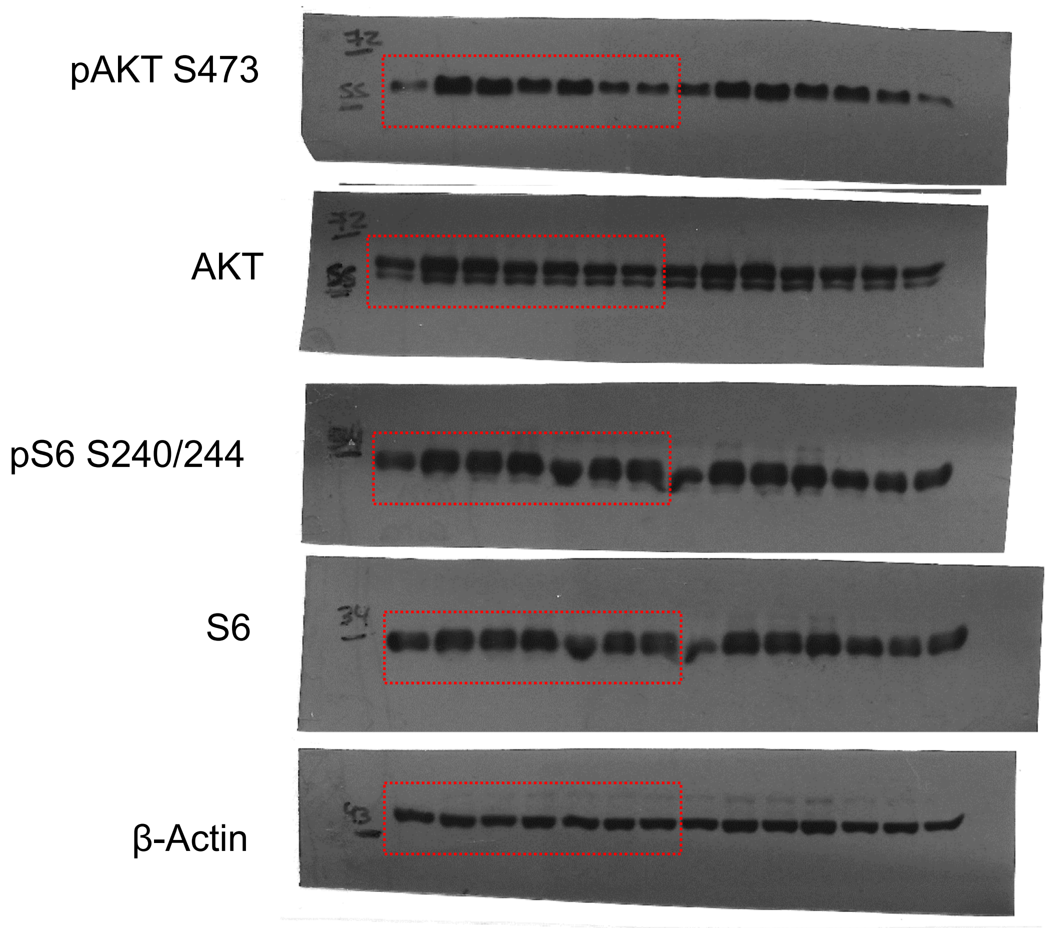

Supplementary Figure 2c

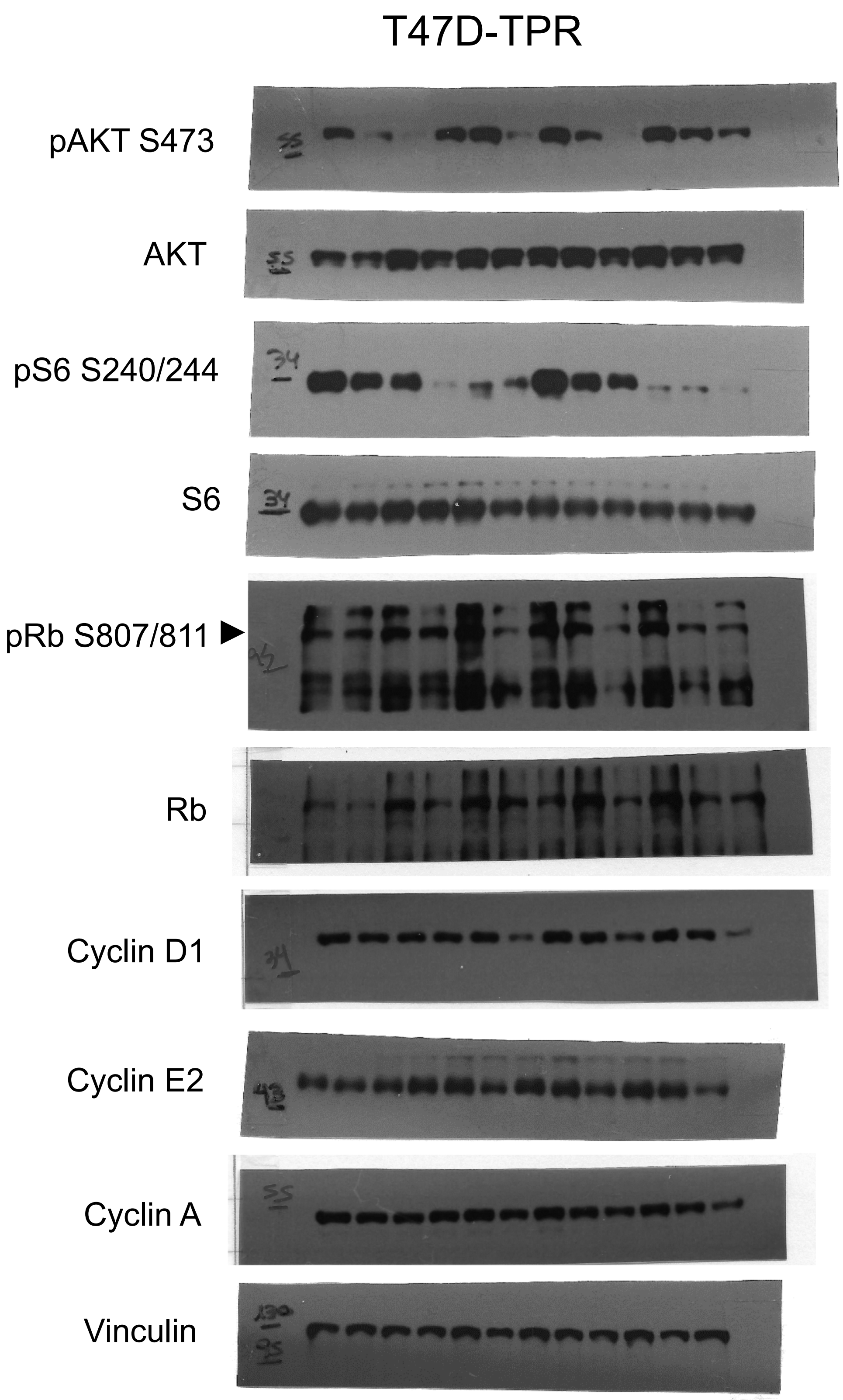

Supplementary Figure 2e

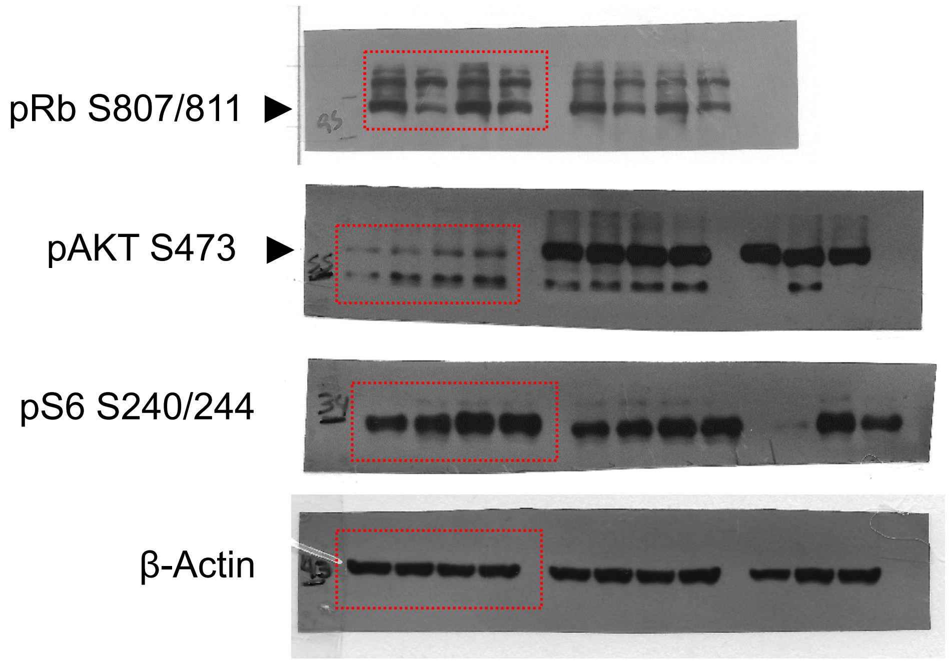

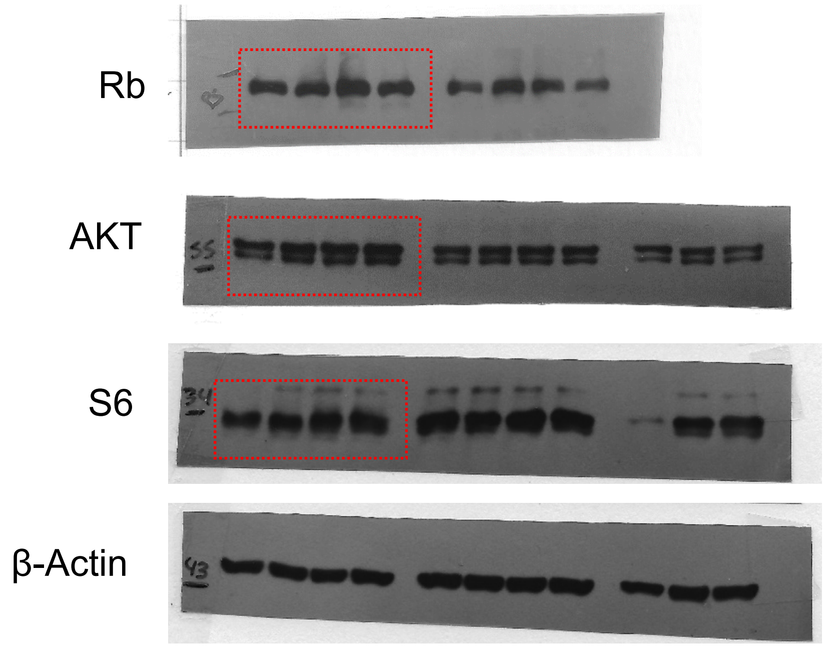

Supplement: Supplementary file 3 — Supplementary Information 3. [file 41598_2023_29425_MOESM3_ESM.pdf]
